# Exogenous gibberellic acid shortening after-ripening process and promoting seed germination in a medicinal plant *Panax notoginseng*

**DOI:** 10.1186/s12870-023-04084-3

**Published:** 2023-02-01

**Authors:** Na Ge, Jin-Shan Jia, Ling Yang, Rong-Mei Huang, Qing-Yan Wang, Cui Chen, Zhen-Gui Meng, Long-Geng Li, Jun-Wen Chen

**Affiliations:** 1 College of Agronomy and Biotechnology, Yunnan Agricultural University, Kunming, 650201 Yunnan China; 2 The Key Laboratory of Medicinal Plant Biology of Yunnan Province, Yunnan Agricultural University, Kunming, 650201 Yunnan China; 3 National and Local Joint Engineering Research Center on Germplasm Innovation and Utilization of Chinese Medicinal Materials in Southwestern China, Yunnan Agricultural University, Yunnan 650201 Kunming, China

**Keywords:** After-ripening process, Gibberellic acid, Morphophysiological dormancy, *Panax notoginseng*, Recalcitrant seeds, Transcriptome

## Abstract

**Background:**

*Panax notoginseng* (Burk) F.H. Chen is an essential plant in the family of Araliaceae. Its seeds are classified as a type of morphophysiological dormancy (MPD), and are characterized by recalcitrance during the after-ripening process. However, it is not clear about the molecular mechanism on the after-ripening in recalcitrant seeds.

**Results:**

In this study, exogenous supply of gibberellic acid (GA_3_) with different concentrations shortened after-ripening process and promoted the germination of *P. notoginseng* seeds. Among the identified plant hormone metabolites, exogenous GA_3_ results in an increased level of endogenous hormone GA_3_ through permeation. A total of 2971 and 9827 differentially expressed genes (DEGs) were identified in response to 50 mg L^−1^ GA_3_ (LG) and 500 mg L^−1^ GA_3_ (HG) treatment, respectively, and the plant hormone signal and related metabolic pathways regulated by GA_3_ was significantly enriched. Weighted gene co-expression network analysis (WGCNA) revealed that GA_3_ treatment enhances GA biosynthesis and accumulation, while inhibiting the gene expression related to ABA signal transduction. This effect was associated with higher expression of crucial seed embryo development and cell wall loosening genes, *Leafy Contyledon1* (*LEC1*), *Late Embryogenesis Abundant* (*LEA*), *expansins* (*EXP*) and *Pectinesterase* (*PME*).

**Conclusions:**

Exogenous GA_3_ application promotes germination and shorts the after-ripening process of *P. notoginseng* seeds by increasing GA_3_ contents through permeation. Furthermore, the altered ratio of GA and ABA contributes to the development of the embryo, breaks the mechanical constraints of the seed coat and promotes the protrusion of the radicle in recalcitrant *P. notoginseng* seeds. These findings improve our knowledge of the contribution of GA to regulating the dormancy of MPD seeds during the after-ripening process, and provide new theoretical guidance for the application of recalcitrant seeds in agricultural production and storage.

**Supplementary Information:**

The online version contains supplementary material available at 10.1186/s12870-023-04084-3.

## Background

The seed is the most critical period in the life cycle of a plant [1]. Seed dormancy is traditionally defined as an intrinsic obstacle to germination under a favorable condition [2–4]. It is crucial for the conservation of germplasm resources, and the prevention of pre-harvest sprouting [5]. The morphological features of seed-covering tissues and the physiological status of the embryo are an essential determinant of seed dormancy [6], and it is classified as physiological (PD), morphological (MD), physical (PY), combinational (PY + PD) and morpho-physiological (MPD) types [7, 8]. The MPD seeds have to undergo an after-ripening (AR) process characterized by a gradual reduction in dormancy level [9]. The seeds with the after-ripening process would undergo an intricate range of metabolic processes before germination, which is in preparation for the mobilization of food reserve and cell growth [10]. The after-ripening process depends on moisture and oil contents, seed-covering structures and temperature [11]. With a low level of dormancy, temperatures and light could overcome the final limitations on germination [12], and germination happens if water potential is adequate to allow the radicle to protrude [13]. The after-ripening is a process in which the dormancy is lost in a low-hydrated state. However, it remains largely unknown about the mechanism of dormancy release of MPD seeds that show a high water content during the after-ripening process.

The elicitation, retention and reduction of seed dormancy is a highly intricate physiological process that relies on a multitude of endogenous and environmental factors [14]. Signals from hormones, essentially those of abscisic acid (ABA) and gibberellin (GA) are integrators between environmental cues and molecular signals, thus regulating gene expression [9, 15]. The balance of GA and ABA is a critical factor in controlling seed dormancy and germination [4]. Fluctuating temperatures enhance the ratio of GA/ABA by decreasing ABA content in *Cynara cardunculus* seeds, and simultaneously the expression of *Nine-cis-epoxycarotenoid dioxygenase* (*NCED*) and *ABA-INSENSITIVE5* (*ABI5*) is inhibited [16]. In the *Arabidopsis* and tomato (*Lycopersicon esculentum* M.), the mutants with defects in the gene encoding GA biosynthetic enzymes are unable to germinate [17]. The dormancy loss in wheat seeds is accompanied by the increased expression of *TaGA20ox* and the enhanced level of bioactive GA_1_ during imbibition [18, 19]. *Arabidopsis* plants constitutively expressing GA catalytic enzyme GA 2-oxidase (GA2ox) reveal that the reduced accumulation of GA in seeds leads to the increased probability of seed abortion [20]. The seed germination depends on gibberellin (GA) and is inhibited by DELLA when GA concentration is relatively low in *Arabidopsis*. Seeds of GA-deficient mutant *ga1* (*GA Requiring 1*) exhibit a failure to germinate phenotype in the lack of exogenous gibberellic acid (GA_3_) [21]. In contrast, the mutant *GA2ox* could deactivate bioactive GA, consequently accompanying a decreased level of seed dormancy [22]. Overall, the signaling and content of ABA and GA play a critical role in regulating seed dormancy.

GA is required in seed development, and exogenous GA_3_ has been applied to break seed dormancy. Exogenous GA_3_ facilitates the germination of *Fraxinus hupehensis* seeds by enhancing the level of soluble sugars and weakening lipolysis [23]*.* Similarly, exogenous GA_3_ might increase starch hydrolysis by stimulating the catalytic activity to mitigate oxidative damages in the early germination of *Zanthoxylum dissitum* seeds [24]. It has been found that exogenous GA_3_ application prompts GA signal transduction and suppresses ABA synthesis to facilitate rice seed germination under low-temperature conditions [25]. Exogenous GA_3_ might break seed dormancy and promote seed germination. However, little information is available about the response of dormancy release in recalcitrant seed to exogenous GA_3_.

*Panax notoginseng* (Burkill) F. H. Chen (Sanqi in Chinese), a traditional Chinese medicinal plant, is a perennial herb from the family of Araliaceae [26]. Its seeds are classified into the group of morphophysiological dormancy (MPD). Moreover, it has been characterized by the typically recalcitrant trait [27, 28], the water content is about 67.3% in seeds at morphological maturation, and the seeds are highly sensitive to dehydration, the viability of seeds under natural conditions is only 15 days [29, 30]. Most recalcitrant seeds might quickly germinate after shedding, but some recalcitrant seeds with incomplete development of embryo have to undergo the after-ripening process before the germination [31]. Among of them, *P. notoginseng* seeds need to undergo about 45 ~ 60 days of after-ripening process before germination [32]. A preliminary study has demonstrated that incompletely developed embryos might result in the dormancy of *P. notoginseng* seed, the embryo in the postharvest seed at a heart-shaped period has to be further differentiated and developed during the after-ripening process [33]. Meanwhile, soluble sugar, starch and protein gradually decrease with the prolonged storage time in the seeds of *P. notoginseng* during the after-ripening process [34, 35]. The lack or inadequate accumulation of LEA proteins in the embryo tissues and the low activity of GSH metabolism might be the key factors leading to the dehydration sensitivity in recalcitrant seeds of *P. notoginseng* [36]. *PE2*, *GAI*, *KS*, *PP2C*, *GA2OX* and other genes have been identified as the key genes involved in regulating the dormancy release of *P. notoginseng* seeds [37]. Our recent work has shown that exogenous GA_3_ treatment could effectively shorten the after-ripening process and stimulate seed germination of *P. notoginseng* [38]. However, it is still unknown about the mechanisms through that GA_3_ facilitate the germination of postharvest *P. notoginseng* seeds at the physiological and molecular levels. In this study, we compared germination rate, hormonal content, and transcriptomic-related indicators of seeds treated with exogenous GA_3_. We also identified differentially expressed genes (DEGs) associated with seed germination and the expression of genes related to seed germination verified by qRT-PCR. Our data revealed the physiological and transcriptomic aspects of the promoted effect of GA_3_ on recalcitrant seed germination.

## Results

### Effects of exogenous GA_3_ treatment on physiological indexes during *P. notoginseng* seed after-ripening process

**Fig. 1:**
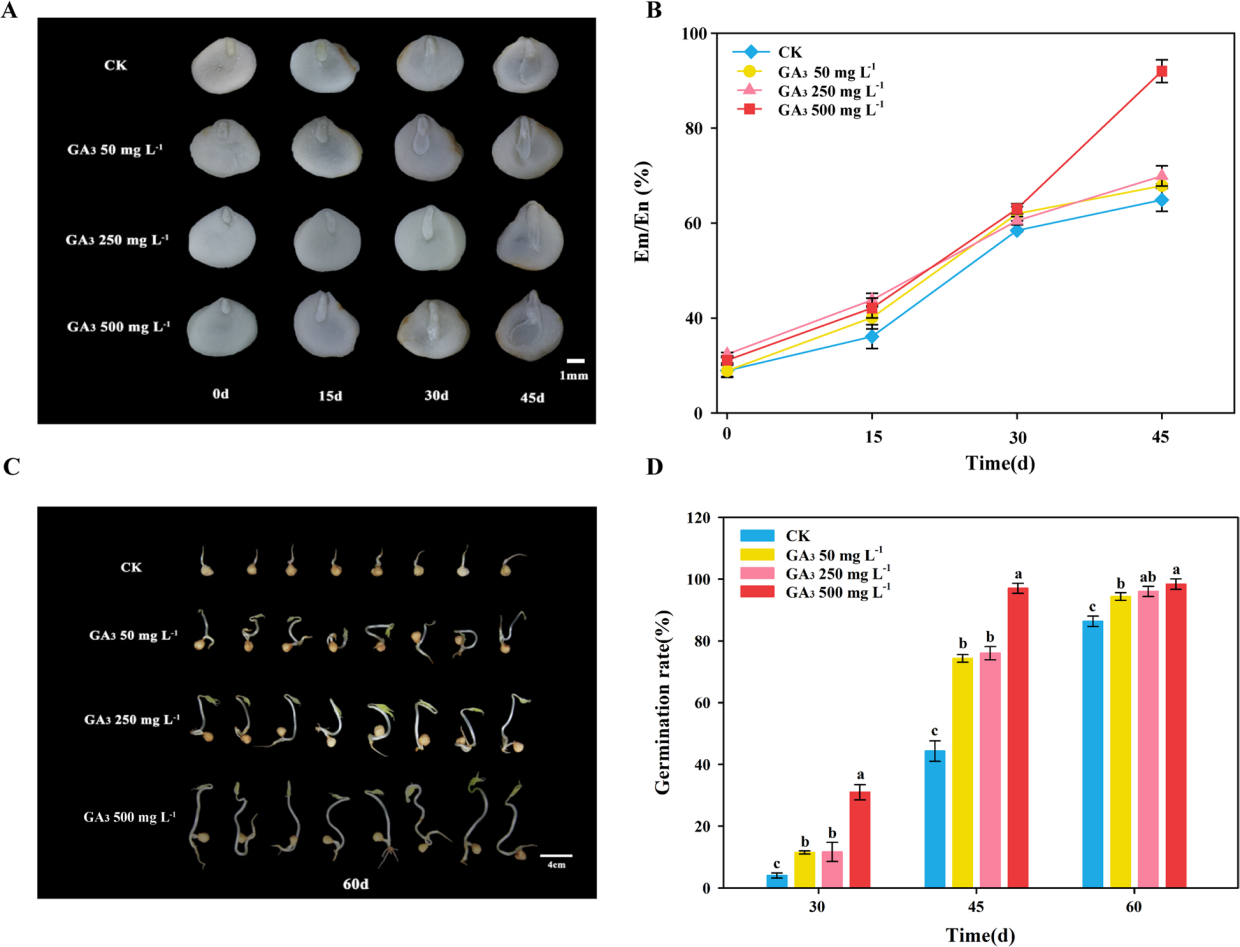
Application of exogenous GA_3_ to promote seed germination of *P. notoginseng*. **A** Appearance and stereoscopic micrographs of control and GA_3_-treated *P. notoginseng* seeds during the after-ripening process. **B** Changes in Em/En ratio of control and GA_3_-treated *P. notoginseng* seeds during the after-ripening process. **C** Appearance and morphology of control and GA_3_-treated *P. notoginseng* seeds after germination (t = 60d). **D** Changes in the rate of germination in control and GA_3_-treated *P. notoginseng* seeds during the after-ripening process. The values presented are the means ± SE (*n* = 3). Different letters indicate significant differences among treatments in the same period using Duncan’s test (*P* < 0.05)

In this study, the seeds were obtained by sandy stratification after treatment with 50 mg L^−1^ GA_3_ (LG), 250 mg L^−1^ GA_3_ (MG), 500 mg L^−1^ GA_3_ (HG) and water (CK). Embryo development and seed germination were shown in Fig. 1. At 0 DAR, the embryos were enclosed by endosperms (Fig. 1A). The size of the embryos generally increased as the after-ripening process was prolonged (Fig. 1A-B). The embryo length was more than half of the seeds sections at 30 DAR, and the rates of Em/En of 50 mg L^−1^, 250 mg L^−1^and 500 mg L^−1^ GA_3_-treated seeds were 61.98%, 60.50% and 63.01%, respectively. At 45 DAR, the 500 mg L^−1^ GA_3_-treated seeds had a significantly higher Em/En rate (92%) than 50 mg L^−1^ GA_3_-treated seeds and the control (Fig. 1B). Beginning 30 days after treatment, GA_3_-treatment significantly enhanced the germination rate of *P. notoginseng* seeds (Fig. 1D). Compared with 30 DAR, the seed germination rate in control was raised by 10.0% and 27.0% at 45 DAR and 60 DAR, respectively, while the increase in the 50 mg L^−1^ GA_3_-treated seeds were 30.0% and 53.0%, respectively, during the same period (Fig. 1D). Compared with CK, the external application of GA_3_ significantly promoted seed germination of *P. notoginseng*, the rate of seed germination tended to raise as the GA_3_ application increased (Fig. 1C, Fig. S1).

### RNA sequencing and gene annotation of *P. notoginseng* seed transcriptome

To acquire a general overview of the regulation in seed germination as impacted by exogenous GA_3_ treatment with different concentrations, the samples from the concentrations of 50 mg L^−1^ (LG), 500 mg L^−1^ (HG) of GA_3_ and CK at each sampling point (0, 30, 50 DAR) were selected based on the results from experiments (Fig. 1) for transcriptome assays. 27 cDNA libraries from the whole seed were selected to examine the transcriptome level of gene expression in *P. notoginseng* seeds. A total of 187.75 gigabytes (Gb) clean sequencing data was acquired (Table S1). For each sample, the average clear data was about 6.95 Gb, and Q30 (the percentage of bases with Phred > 30 to the total bases, Phrede = -10log_10_(e)) was about 94%. The data were used for comparisons with the reference genome of *P. notoginseng* [39]. 86.09% ~ 88.82% of the reads in the 27 libraries were uniquely mapped by alignment with the reference genome of *P. notoginseng* (Table 1). The transcript abundances of genes were assessed by fragments per kilobase of exon per million fragments mapped (FPKM). The distribution of the log_2_ (FPKM + 1) showed relatively high gene expression as shown in Fig. S2. In this experiment, all R^2^ values between the three biological replicates were closer to 1 by using the Persons Correlation Coefficient (R) (Fig. S3), showing that the biological replicates of each sample had strong correlations.

**Table 1:** Comparative genome statistics of samples

Sample	Total reads	Total map	Unique map	Multi map	Mapping rate (%)
CK-0–1	46,815,702	42,910,019	40,899,591(87.36%)	2,010,428(4.29%)	91.66%
CK_0_2	46,919,124	42,880,938	40,775,136(86.91%)	2,105,802(4.49%)	91.39%
CK_0_3	45,916,162	42,104,871	40,017,119(87.15%)	2,087,752(4.55%)	91.70%
LG_0_1	46,769,434	42,497,124	40,923,834(87.5%)	1,573,290(3.36%)	90.87%
LG_0_2	44,727,404	40,601,250	39,100,103(87.42%)	1,501,147(3.36%)	90.77%
LG_0_3	43,111,468	39,537,923	37,614,568(87.25%)	1,923,355(4.46%)	91.71%
HG_0_1	46,684,610	42,938,303	40,777,893(87.35%)	2,160,410(4.63%)	91.98%
HG_0_2	45,715,822	41,919,574	39,764,745(86.98%)	2,154,829(4.71%)	91.70%
HG_0_3	51,812,850	47,721,232	45,354,018(87.53%)	2,367,214(4.57%)	92.10%
CK_30_1	46,770,276	43,229,185	41,469,671(88.67%)	1,759,514(3.76%)	92.43%
CK_30_2	46,087,118	42,530,822	40,909,769(88.77%)	1,621,053(3.52%)	92.28%
CK_30_3	50,775,950	46,563,721	44,146,740(86.94%)	2,416,981(4.76%)	91.70%
LG_30_1	44,439,024	41,026,549	39,462,059(88.8%)	1,564,490(3.52%)	92.32%
LG_30_2	46,805,200	43,226,074	41,570,186(88.82%)	1,655,888(3.54%)	92.35%
LG_30_3	44,743,018	40,935,834	39,281,645(87.79%)	1,654,189(3.7%)	91.49%
HG_30_1	48,011,460	44,131,637	42,367,279(88.24%)	1,764,358(3.67%)	91.92%
HG_30_2	47,257,916	43,528,792	41,720,947(88.28%)	1,807,845(3.83%)	92.11%
HG_30_3	46,156,508	42,433,078	40,956,718(88.73%)	1,476,360(3.2%)	91.93%
CK_50_1	46,450,880	41,971,359	39,989,166(86.09%)	1,982,193(4.27%)	90.36%
CK_50_2	44,037,090	40,404,986	38,490,509(87.4%)	1,914,477(4.35%)	91.75%
CK_50_3	49,358,510	45,429,117	43,281,381(87.69%)	2,147,736(4.35%)	92.04%
HG_50_1	44,787,148	40,771,025	39,010,023(87.1%)	1,761,002(3.93%)	91.03%
HG_50_2	48,294,500	44,388,897	42,618,190(88.25%)	1,770,707(3.67%)	91.91%
HG_50_3	42,958,006	39,350,379	37,781,609(87.95%)	1,568,770(3.65%)	91.60%
LG_50_1	45,946,054	42,287,132	40,654,703(88.48%)	1,632,429(3.55%)	92.04%
LG_50_2	45,088,374	41,649,863	40,047,536(88.82%)	1,602,327(3.55%)	92.37%
LG_50_3	45,204,254	41,657,387	39,812,720(88.07%)	1,844,667(4.08%)	92.15%

### Comparative analysis of DEGs in *P. notoginseng* seeds with exogenous GA_3_ treatment

**Fig. 2:**
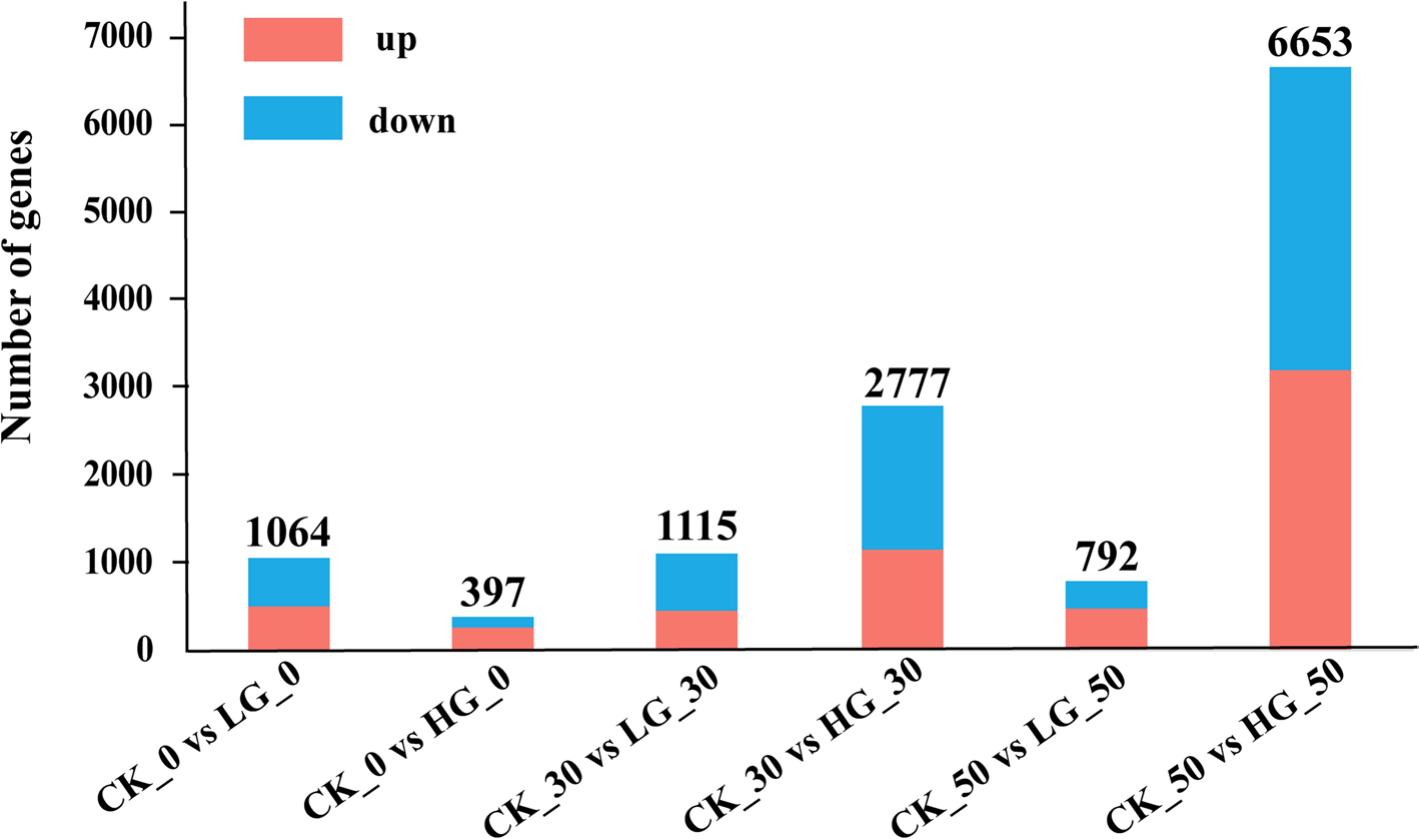
Statistical analysis of differentially expressed genes (DEGs) in control and GA_3_-treated *P. notoginseng* seeds during the after-ripening process

**Fig. 3:**
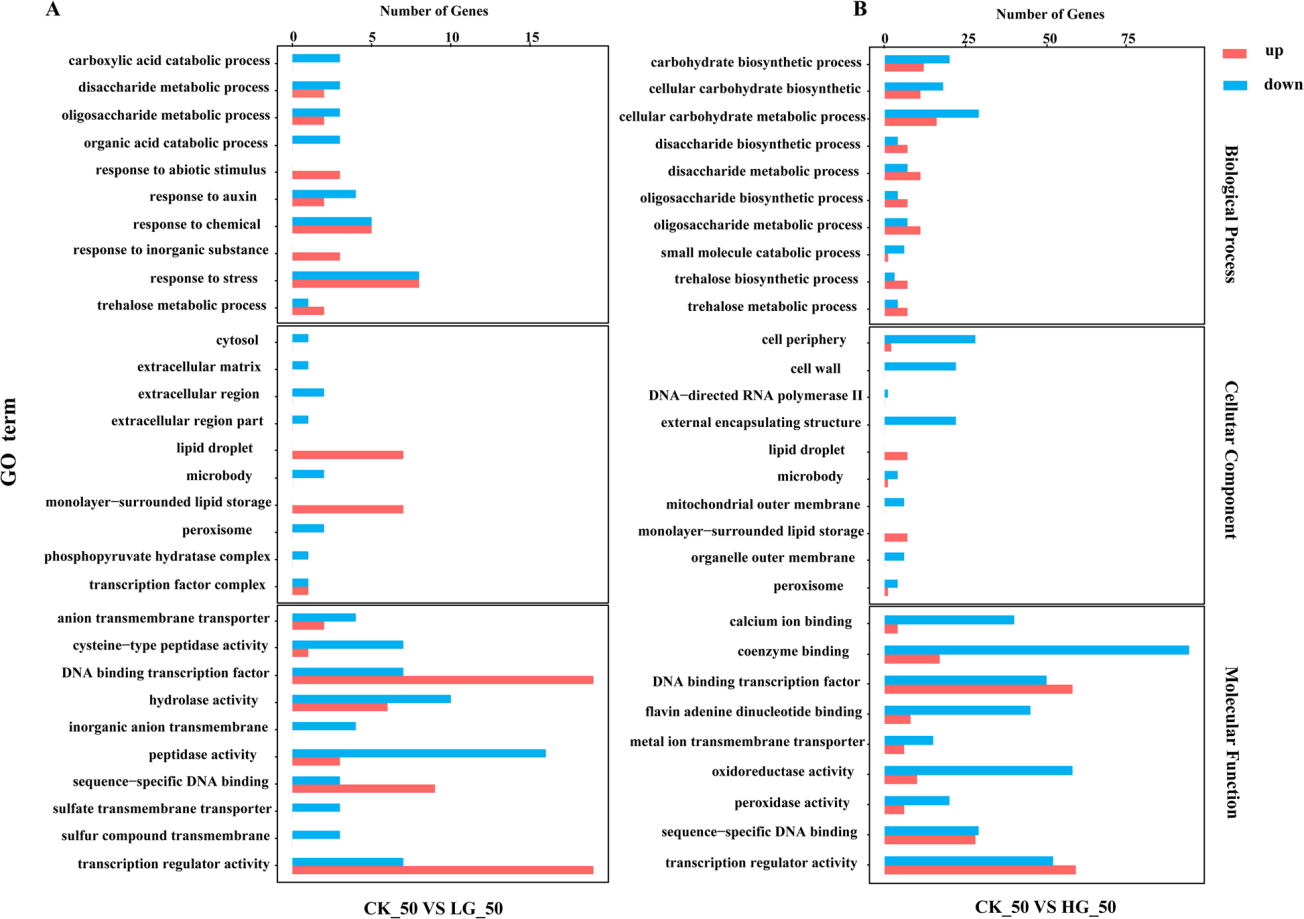
GO terms of differentially expressed genes (DEGs) in control and GA_3_-treated *P. notoginseng* seeds during after-ripening process. **A** DEGs between CK_50 VS LG_50. **B** DEGs between CK_50 VS HG_50. The Y-axis on the left represents GO terms, including biological process, cellular component, and molecular function, the X-axis indicates genes number of each term. Up-regulated genes are shown in the red bar, and down-regulated genes are shown in the blue bar

DEGs were analyzed using the FPKM method to determine the degree of overlap between the three seed groups. Compared with CK, a total of 2971 and 9827 DEGs were identified in *P. notoginseng* seeds with exogenous LG and HG treatment, respectively (Fig. S4). Through pairwise comparisons, a total of 1064, 397, 1115, 2777, 792 and 6653 DEGs were identified at CK_0 d vs LG_0 d, CK_0 d vs HG_0 d, CK_30 d vs LG_30 d, CK_30 d vs HG_30 d, CK_50 d vs LG_50 d and CK_50 d vs HG_50 d, respectively (Fig. 2). In HG-treated seeds at 0 DAR, 265 were up-regulated and 132 down-regulated. At 30 DAR, 1144 genes were up-regulated and 1633 genes were down-regulated on the CK compared with HG-treated seeds (Fig. 2). At 50 DAR, 474 genes were up-regulated and 318 genes were down-regulated on the CK compared with LG-treated seeds (Fig. 2); 3187 genes were up-regulated and 3466 genes were down-regulated on the CK compared with HG-treated seeds. To obtain the functional annotations of DEGs, GO annotation analysis was performed on DEGs (Fig. 3). The results showed that most DEGs were enriched in biological process and molecular function, while a number of DEGs were enriched in the cellular components in the comparisons at CK_30 d vs LG_30 d, CK_30 d vs HG_30 d, CK_50 d vs LG_50 d and CK_50 d vs HG_50 d (Fig. 3, Fig. S5). In biological process classification, these DEGs were specifically involved the metabolic processes, cells biological processes, and response the stress and abiotic stimulus. The molecular functions mainly included binding, catalysis, and transport activity. Besides, most of the gene products were located in cells and organelles.

**Fig. 4:**
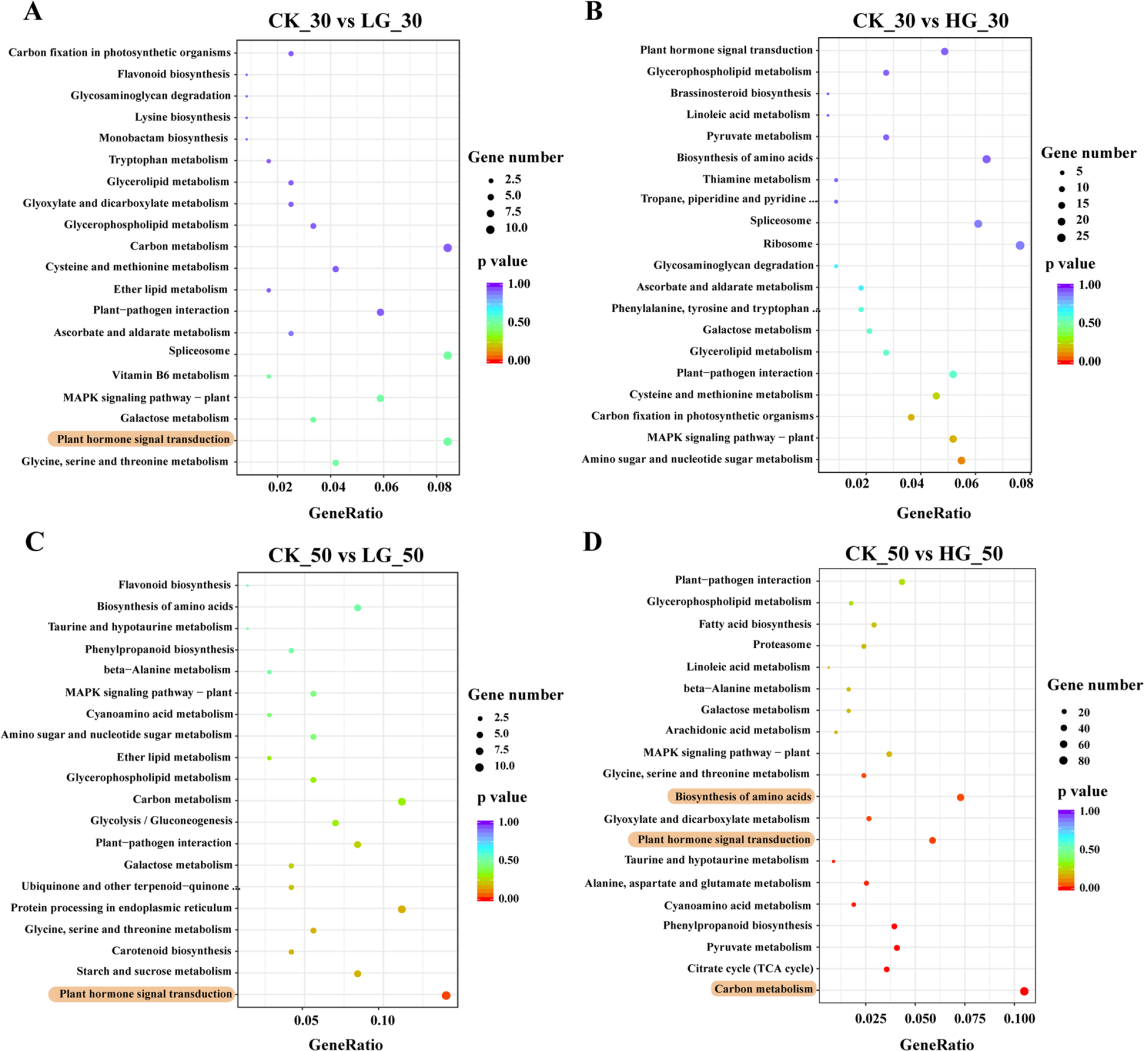
KEGG analysis of differentially expressed genes (DEGs) in control and GA_3_-treated *P. notoginseng* seeds during the after-ripening process. **A** DEGs between CK_30 vs LG_30. **B** DEGs between CK_30 vs HG_30. **C** DEGs between CK_50 vs LG_50. **D** DEGs between CK_50 vs HG_50. The Y-axis on the left represents GO KEGG pathways, the X-axis indicates the “Gene Ratio” represented by the ratio of DEGs numbers to the total annotated gene numbers of each pathway. Low *P* values are shown in the red circle, and high *P* values are shown in the purple circle. The area of a circle represents DEGs number

Moreover, KEGG analysis was used to evaluate the biological functions of the DEGs (Fig. 4). In the comparisons at CK_0 d vs LG_0 d and CK_0 d vs HG_0 d, and the DEGs were mainly identified in pentose and glucuronate interconversions, cyanoamino acid metabolism and protein processing in the endoplasmic reticulum (Fig. S6). In the comparisons at CK_30 d vs LG_30 d and CK_30 d vs HG_30 d, our results found that DEGs were mainly identified in plant hormone signal transduction, galactose metabolism, and amino sugar and nucleotide sugar metabolism. Importantly, in the comparisons at CK_50 d vs LG_50 d and CK_50 d vs HG_50 d, the results showed that the plant hormone signal transduction, carbon metabolism and citrate cycle were enriched in top KEGG pathways (Fig. 4C). Thus, DEGs functional enrichment suggested that plant hormone signal transduction and carbon metabolism were closely involved in GA_3_-promoted seed development, and these pathways were further investigated.

### Changes in profiles of plant hormone signal transduction and related metabolites in response to exogenous GA_3_ treatment

**Fig. 5:**
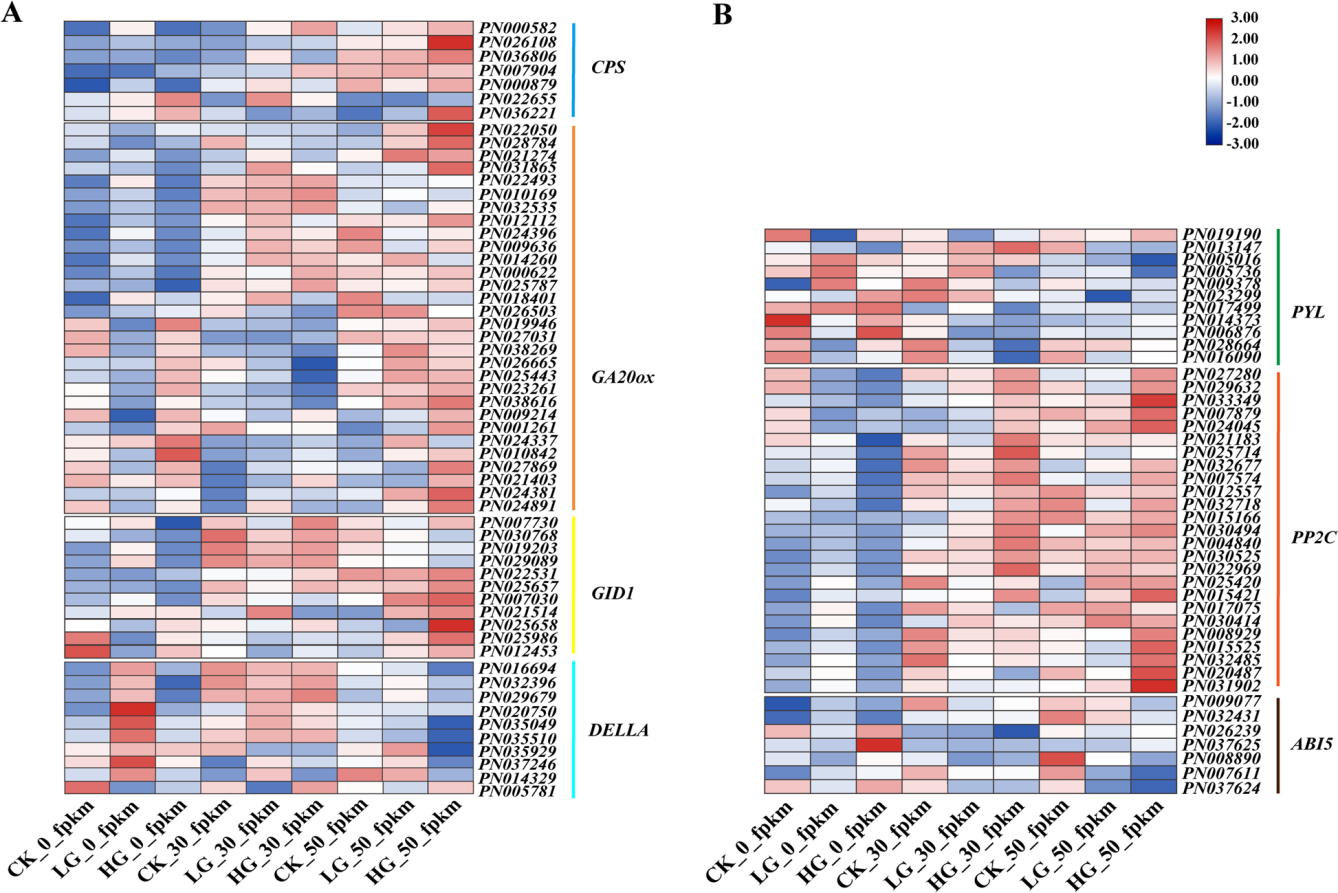
Expression pattern analysis base on RNA-seq of GA (**A**) and ABA (**B**) biosynthesis and signal transduction pathway-related genes of *P. notoginseng* seeds treated with GA_3_ during the after-ripening process. In the heat map, different color indicates the expression level changes in GA_3_-treated seeds compared with the control during the after-ripening process

The KEGG annotations revealed that DEGs were related to the plant hormone signal transduction pathway in *P. notoginseng* seeds. To identify highly correlated DEGs that responded to exogenous GA_3_ treatment, the map showed the expression pattern of DEGs related to plant hormone ABA and GA signal transduction (Fig. 5). Compared with the CK, GA_3_ treatment up-regulated DEGs related to GA biosynthesis and signal transduction during the after-ripening process, including *ent-copalyl diphosphate synthase* (*CPS*), *GA20-oxidase* (*GA20ox*) and *GA INSENSITIVE DWARF1* (*GID1*), whereas down-regulated *DELLA*. Further, GA_3_-treatment down-regulated most of the DEGs involved in ABA transport signal transduction pathway among them are *Pyrabactin resistance 1-like* (*PYL*) and *ABSCISIC ACIDINSENSITIVE 5* (*ABI5*), whereas up­regulated *Protein Phosphatase 2C* (*PP2C*). To explore the functions of endogenous hormones in seed germination, ABA, GA_3_ and IAA contents in the CK and GA_3_-treated *P. notoginseng* seeds during the after-ripening process were detected using LC–MS (Table 2). Compared with the CK, endogenous hormones GA_3_ content was significantly the highest at 0 DAR, but IAA content was not changed considerably in the GA_3_-treated group. By contrast, ABA contents were the lowest in the GA_3_-treated group. Thus, GA_3_ treatment could stimulate seed germination by altering the accumulation of endogenous hormones. Moreover, exogenous GA_3_ induced seed germination through increased GA_3_ concentration and decreased ABA concentration. These results imply that exogenous GA_3_ enhances GA biosynthesis and accumulation, while inhibiting the gene expression related to ABA signal transduction.

**Table 2:** Changes of endogenous hormones in the control and GA_3_-treated *P. notoginseng* seeds during after-ripening process

Endogenous hormone (ng/g)	Time (d)	Sample		
		CK	GA_3_ 50 mg/L	GA_3_ 500 mg/L
GA_3_	0	0.432 ± 0.078	4.006 ± 0.358	27.851 ± 2.118*
	30	0.197 ± 0.059	2.914 ± 2.582	15.118 ± 2.277*
	50	0.293 ± 0.244	0.788 ± 0.073	9.100 ± 0.948*
ABA	0	0.250 ± 0.045	0.228 ± 0.044	1.050 ± 0.474
	30	0.154 ± 0.013	0.154 ± 0.023	0.182 ± 0.016
	50	0.116 ± 0.026	0.105 ± 0.007	0.102 ± 0.007
IAA	0	20.465 ± 1.548	19.608 ± 1.091	19.756 ± 1.652
	30	12.695 ± 1.106	13.513 ± 2.230	11.191 ± 0.930
	50	7.108 ± 2.390	7.812 ± 1.471	8.167 ± 1.464

The values are means ± SD(*n* = 3)

^*^P < 0.05, significant differences between the control and GA_3_ treatments

### WGCNA analysis the expression of key genes in response to GA_3_ treatment

**Fig. 6:**
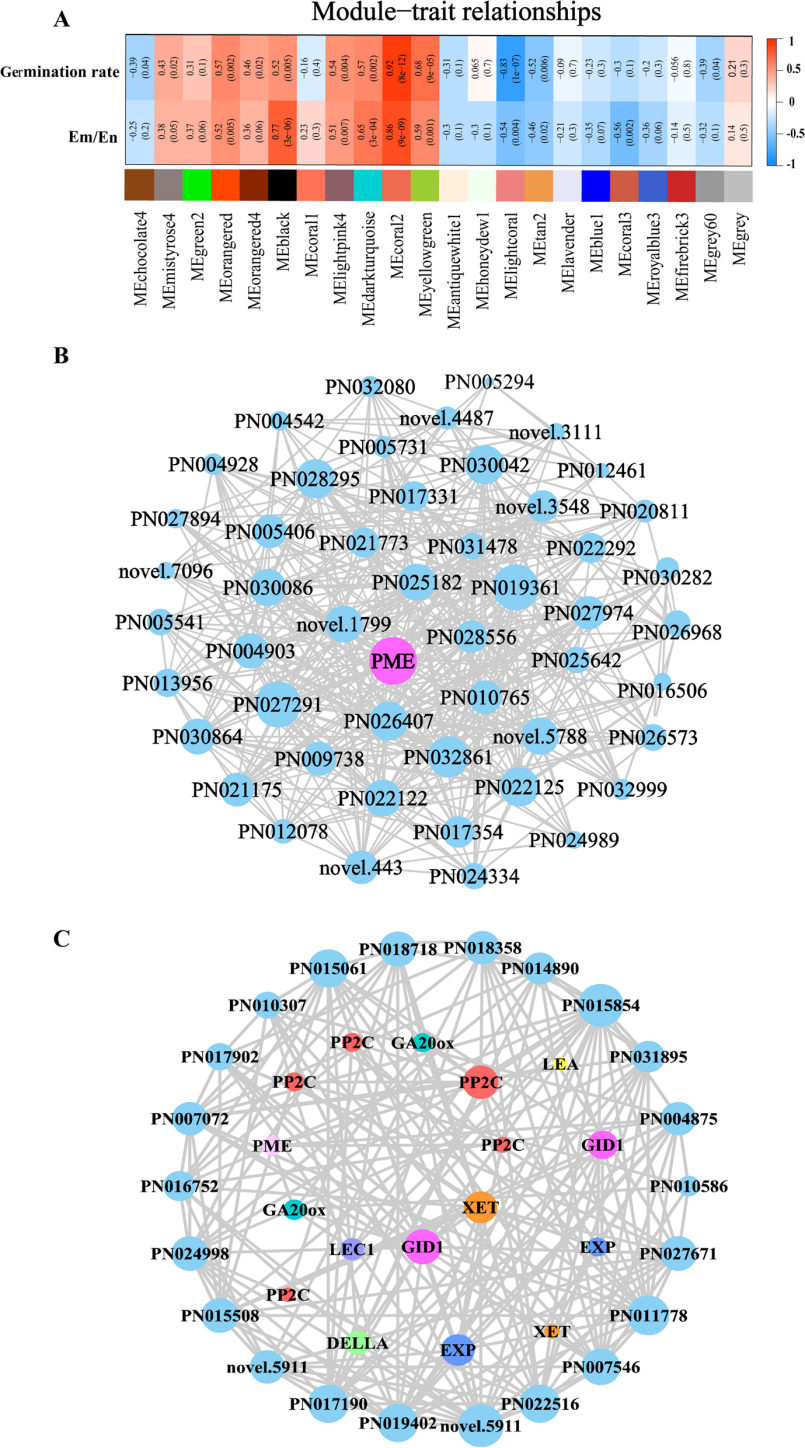
Network analysis of co-expression modules using WGCNA. (**A**) Heatmap of module-trait weight correlations and corresponding *P*-values. (**B**) Potential regulatory network of DEGs in coral2 and black (**C**) modules. Degree indicates the number of lines owned by a node. A line connects two different genes

In order to identify highly correlated genes that co-occurred and responded to exogenous GA_3_ treatment in *P. notoginseng* seeds during the after-ripening process, the Weighted Gene Co-Expression Network Analysis (WGCNA) was performed to analyze gene expressions at 0, 30 and 50 DAR. After removal of the genes with low fluctuation in expression (standard deviation ≤ 0.4), 21,988 of 45,737 genes were subjected to pairwise correlation analysis regarding gene expression and sorted into different twenty-two modules, the genes in the same modules shared high correlation coefficients (Fig. S7). Based on the finding that seed embryos elongated significantly after gibberellin treatment (Fig. 1), the modules significantly associated with the traits were identified. Two modules showed a higher correlation (*r* = 0.86 and *r* = 0.77 for coral2 and black, respectively) with GA_3_ treated, indicating that genes in these modules regulate embryo development and seed germination (Fig. 6A). A total of 2086 and 3732 genes were found in the “coral2” and “black” (*P* < 0.01) modules, respectively. Based on the connectedness of genes, the hub genes *PME* and *PP2C* were screened in coral2 and black modules, respectively (Fig. 6B-C). Moreover, the genes with the higher connectivity included those GA biosynthesis and catabolism (*GID1*, *GA20ox*, *DELLA*), embryo development (*LEA*, *LEC1*) and cell wall loosening (*EXP*, *XET*). It is revealed that these genes may be the key genes of the “coral2” and “black” modules and involved in GA_3_ regulation of *P. notoginseng* seed germination.

**Fig. 7:**
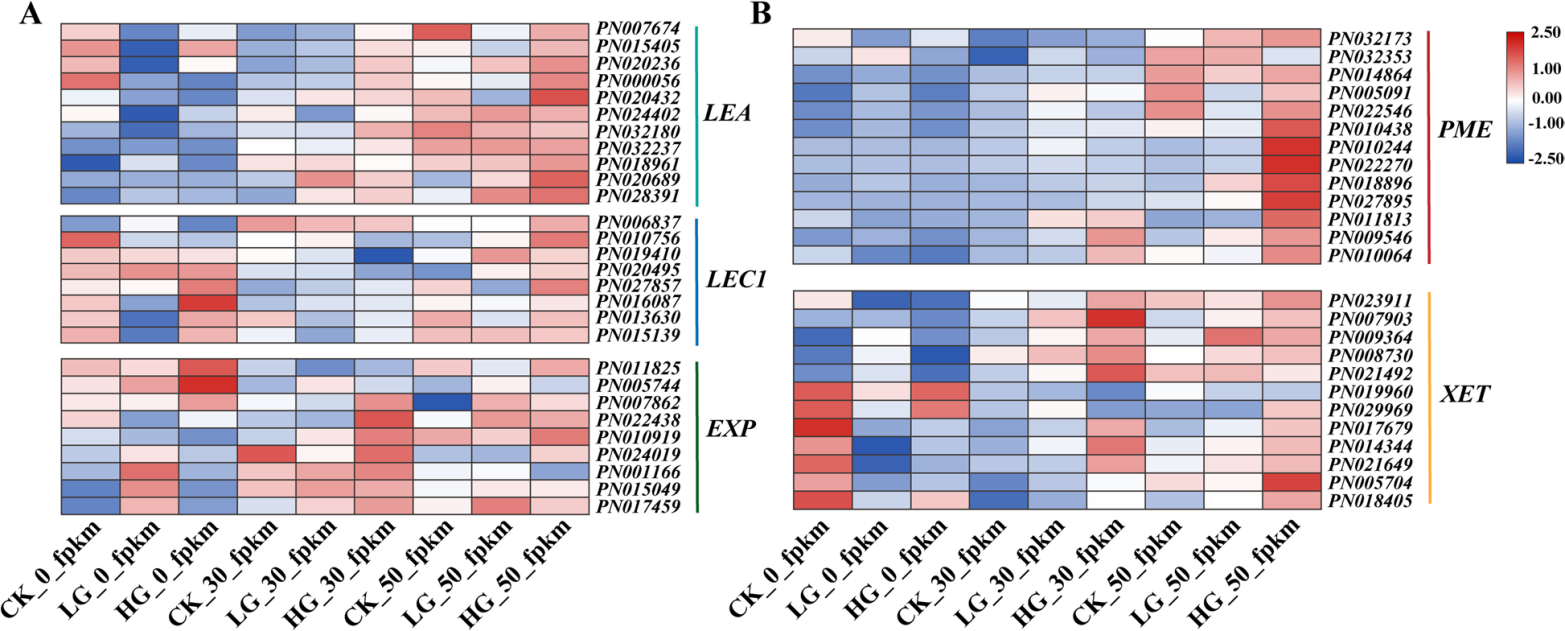
Effects of GA_3_ on profiles of transcriptome of *P. notoginseng* seeds during the after-ripening process. **A** The candidate genes involved in embryo development. **B** The candidate genes involved in cell wall metabolism. In the heat map, different color indicates the expression level changes in GA_3_-treated seeds compared with the control during the after-ripening process

Based on the analysis of WGCNA (Fig. 6), we focused on embryo development-related and cell wall-related DEGs (Fig. 7), which were clustered into five gene groups: *Late Embryogenesis Abundant* (*LEA*), *Leafy Contyledon1*(*LEC1*), *Expansin* (*EXP*), *Xyloglucan Endotransglucosylase* (*XET*) and *Pectin Methylesterase* (*PME*). Compared with the CK, the expression level of *PME* and *LEA* did not change upon GA_3_ treatment at 0 DAR, while GA_3_ increased the expression level of *LEC1* and *EXP*. With the prolonged after-ripening process, *LEA* and *PME* were dramatically up-regulated in response to GA_3_ applications. The expression of *LEC1*, *EXP* and other genes were found to be increased during after-ripening process after GA_3_ treatment.

### Verification of expression of DEGs using qRT-PCR

**Fig. 8:**
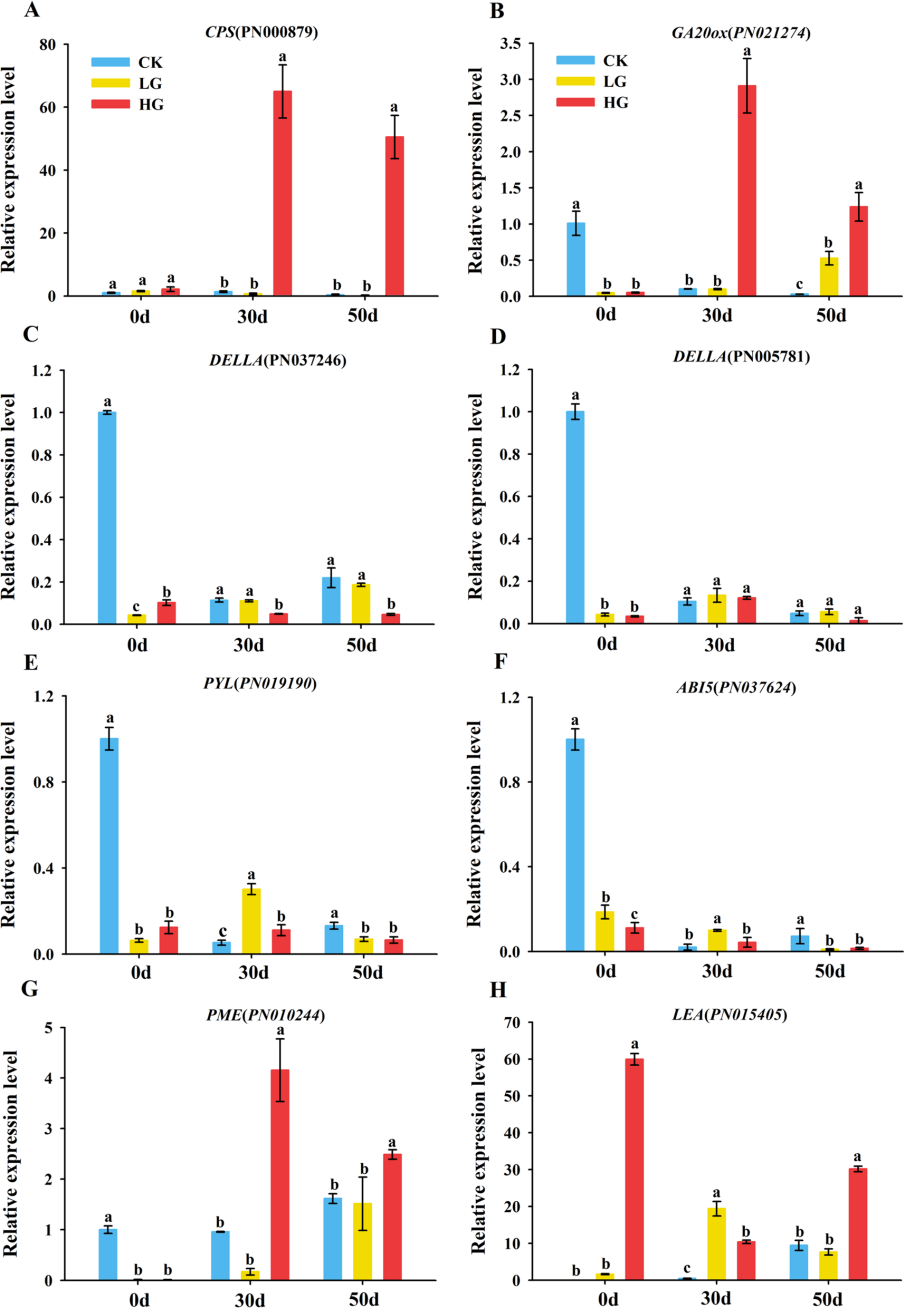
The DEGs expression in response to GA_3_ treatment was analyzed by RT-qPCR. **A** *CPS*, *ent-copalyl diphosphate synthase*. **B** *GA20ox*, *GA20-oxidase*. **C**, **D** *DELLA*. **E** *PYL*, *Pyrabactin resistance 1-like*. **F** *ABI5*, *ABA-INSENSITIVE5*. **G** *PME*, *Pectinesterase*. **H** *LEA*, *Late Embryogenesis Abundant*. The values presented are the means ± SE (*n* = 3). Different letters indicate significant differences among treatments in the same period using Duncan’s test (*P* < 0.05)

To test the reliability and the repeatability of RNA-seq, the DEGs related to GA biosynthesis and signal (*CPS*, *GA20ox*, *DELLA*), ABA signal and response (*PYL*, *ABI5*), embryo development (*LEA*) and cell wall metabolism (*PME*) were chosen for the confirmation of gene expression (Fig. 8). It indicated that the results of transcriptomic were reliable and accurate.

## Discussion

### Exogenous GA_3_ effectively shortens the after-ripening process and promotes seed germination of *P. notoginseng*

Seed dormancy and germination are influenced by plant hormonal and the external environment [40–42]. Exogenous GA_3_ treatment could significantly promote germination in wild species of pistachioo [8, 43]. It has been found that seed germination is substantially facilitated by exogenous GA_3_ treatment in *Fraxinus hupehensis* [23]. Consistently with the finding of previous studies, the present study revealed that exogenous GA_3_ obviously promote seed germination of *P. notoginseng* (Fig. 1C-D). Compared with CK, the seed germination rate tended to be raised as GA_3_ application increased, and the germination rate was highest in *P. notoginseng* seeds treated with 500 mg L^−1^ exogenous GA_3_ (Fig. 1D, Fig. S1). It has also been recorded that *Acer mono* Maxim. seeds are treated with 200 mg L^−1^ GA_3_ and their germination rate is effectively increased [44]. *Nitraria tangutorum* Bobr. seeds are treated with 150 mg L^−1^ GA_3_ and germination rate, the germination index and vigor index are the highest [45]. These studies indicate that the appropriate concentration of exogenous GA_3_ is one of the key factors in breaking dormancy and promoting seed germination. In our research, we verified the impacts of different concentrations of GA_3_ (LG, MG and HG) on seed embryo development, germination rate and after-ripening process of *P. notoginseng* seeds (Fig. 1B-D), and found that the germination rate of *P. notoginseng* seed is gradually elevated with the increase of exogenous GA concentration (Fig. 1, Fig. S1), indicating that 500 mg L^−1^ of exogenous GA_3_ is most appropriate to promote seed germination of recalcitrant *P. notoginseng* seeds.

GA essentially stimulates endosperm weakening and embryo expansion [46], and promotes the protrusion of radicles by breaking through the confines of the seed coat [47, 48]. Herein, we found that the endosperm tissue around seed embryos treated with HG is softened at 30 DAR compared with CK (Fig. 1A), implying that GA_3_ treatment might stimulate the softening of tissues around seed embryo, thus providing sufficient space for embryo development. Our results agreed with the observation that GA accelerates the growth potential of the embryo and weakens the structures surrounding the embryo in tomato [49]. Above all, our results reveal that 500 mg L^−1^ GA_3_ treatment might effectively shorten the after-ripening process and promote seed germination by stimulating seed embryo development and softening the tissues around the embryos of *P. notoginseng.*

### Exogenous GA_3_ application accelerates *P. notoginseng* seed germination by changing endogenous hormone accumulation

ABA and GA antagonistically regulate seed dormancy and germination [40, 50]. The induction and maintenance of dormancy are positively regulated by ABA, while germination is enhanced by GA [51]. Consistently, our results showed that GA_3_ content is the highest, and ABA content is the lowest in *P. notoginseng* seeds treated with GA_3_ (Table 2). This is agreement with other study showing an increase in energy requirements and endogenous GA_3_ content but a decrease in ABA content during germination and growth of seeds [23]. Compared with the CK, our study found that the GA_3_ content in *P. notoginseng* seeds had a 60-fold increase after treatment with 500 mg L^−1^ GA_3_, followed by a ninefold increase in seeds after treatment with 250 mg L^−1^ GA_3_ at 0 DAR (Table 2). The levels were much too high to be endogenous GA_3_ and they were reduced with time after treatment. It could be the result that the penetration of exogenous GA_3_ into the seed tissues was caused by the concentration difference between the soak solution and the cytolymph during the soaking treatment. Those results indicated that a part of measured endogenous GA_3_ is likely to be remaining from the GA_3_ treatment, but both of them contribute to altering the ratio of GA and ABA. Besides, our results found that exogenous GA_3_ application could not cause auxin (IAA) content to be different in *P. notogensing* seeds, and this is contrary to the finding that exogenous GA_3_ increases IAA content in the tiller node of rice (*Oryza sativa* L.) [52], implying that IAA responds diversely in the regulation network of plant development upon GA_3_ treatment. Thus, we consider that exogenous GA_3_ release dormancy to promote seed germination mainly through changing the ratio of GA and ABA.

Cellular ABA and GA levels are controlled by the balance between their biosynthesis and catabolism [53]. Our transcriptomic analysis revealed that a total of 2971 and 9827 DEGs are dramatically affected by exogenous LG and HG treatment, respectively (Fig. 2). Meanwhile, it was significantly enriched for plant hormone signal transduction and related metabolic pathways regulated by GA (Fig. 4C-D), suggesting that GA induces dramatic responses at the transcriptional level. Some candidate genes in GA_3_ and ABA signaling pathways also determine seed germination [50, 54]. In our study, the expression level of *CPS*, *GID1* and most of *GA20ox* were downregulated by GA_3_ treatment at 0 DAR (Fig. 5). This effect gradually weakened and was lost with decreasing levels of GA_3_ in seeds (Table 2), suggesting that high concentrations of GA_3_ in treated seeds might be a negative regulator to suppress GA biosynthesis and signaling by reducing expression of some GA-biosynthesis genes in a homeostasis mechanism (Binenbaum et al., 2018). A study on barley, wheat and rice has shown that *HvGA20ox* is a pivotal gene for regulating seed germination in barley [55]. *OsGA20ox2* and *OsGA2ox3* were essential genes to control seed germination in rice [56], and the mutation *OsGA20ox2* shows a reduced GA level and enhanced seed dormancy [57]. Likewise, our study found that *CPS*, *GA20ox*, *GID1* and *DELLA* genes involved in GA hormone biosynthesis and catabolism pathways are affected by exogenous GA_3_ treatment (Fig. 5). GA_3_ upregulated the expression of *CPS*, *GA20ox* and *GID1*, and downregulated *DELLA* at 30 DAR and 50 DAR (Fig. 8C-D). DELLA is a plant growth suppressor, while GID1 is a receptor for GA_3_. It acts by binding to the GID1 receptor to degrade the DELLA protein in plants [58, 59]. Overall, the expression levels of *GA20ox* and *GID1* were upregulated, and the expression level of *DELLA* was downregulated by GA_3_ treatment during the after-ripening process, thereby perturbing GA_3_ signal transduction in recalcitrant *P. notogensing* seeds.

A comparative analysis of *PP2C* mutants suggests that AtPP2CA is a significant player in seeds [60, 61]. The ABA receptors PYR1/PYL proteins might confer a prominent function in seed ABA responsiveness through regulating PP2C activity [62, 63], and the *pyr1 prl1 prl2 prl4* quadruple mutant shows ABA insensitive the germination [62]. Genetic analysis reveals that ABA-INSENSITIVE 3(ABI3), ABA-INSENSITIVE 4 (ABI4) and ABA-INSENSITIVE 5 (ABI5) are the key transcription factors that confer seed ABA responsiveness [64]. The seeds of *abi5* mutants reduce transcript levels of *Early Methionine-labelled 1* and *6* (*EM1* and *EM6*), which are associated with the germination process [65, 66]. The WGCNA analysis showed that the *PP2C* was hub gene in the black module (Fig. 6), and the expression of *PYL*, *PP2C* and *ABI5* has a significant change in *P. notoginseng* seeds treated with exogenous GA_3_ (Fig. 5C). *PYL* and *ABI5* showed a higher expression level at the 0 DAR. They gradually decreased with the prolonged after-ripening process in *P. notoginseng* seeds. Surprisingly, compared with CK, the expression of *PYL* and *ABI5* tended to decline as the GA_3_ application increased, and it was lowest in *P. notoginseng* seeds treated with 500 mg L^−1^ exogenous GA_3_ at 50 DAR (Fig. 8E-F), and the expression trend of *PP2C* was reversed during the after-ripening process (Fig. 5). Our results suggest that exogenous GA_3_ regulates the essential genes to perturb endogenous GA and ABA biosynthesis and catabolism in *P. notoginseng* seeds. This might partly contribute to the antagonistic action of GA and ABA on seed germination and growth.

### The elevated endogenous hormone GA effectively promotes the expression of genes related to embryo development and cell wall loosening

The synthesis and catabolism of GA_3_ vigorously promote cell division during seed development and germination [67, 68]. A previous study has shown that the incomplete development of embryos could result in seed dormancy of *P. notoginseng* [33]. Recent studies revealed that *LEAFY COTYLEDON 1* (*LEC1*) is a critical regulator of seed development, its loss of function results in a short embryo axis and intolerance to desiccation [69, 70]. Consistently, Late *Embryogenesis Abundant* (*LEA*) and *LEC1* are required for seed maturation and acquisition of desiccation tolerance [71, 72]. In our study, we found that the expression of *LEC1* and *LEA* is lower in CK at 0 DAR, and they are dramatically up-regulated in response of *P. notoginseng* seeds to GA_3_ applications during the after-ripening process (Fig. 7A), demonstrating that the embryo development is relatively vigorous under GA_3_ treatment (Fig. 1A). These results support the view that GA_3_ treatment could promote the embryo development to boost seed germination of postharvest *P. notogensing*.

The architecture of the cell wall is a crucial determinant of plant growth [73]. The dormancy or germination is determined by the balance between the resistance strength of the surrounding tissues and the growth potential of the elongating radicle [74]. There are a series of evidence that GA_3_ could facilitate radicle protrusion by breaking through the mechanical constraints of the seed coat during seed germination [67, 75]. The cell wall-degrading enzymes, such as cellulases, xyloglucan endotransglucosylase-hydrolase (XTH), pectinesterase (PME), expansins (EXP) and hemicellulases, have been proven to contribute to cell wall loosening [73, 76–78]. In our study, DEGs (*PME*, *EXP* and *XTH*) involved in cell wall development were up-regulated by exogenous GA_3_ treatment (Fig. 6, Fig. 7A-B). Our result is consistent with observations that *xyloglucan endotransglucosylase* (*XET*), *xyloglucan endohydrolase* (*XEH*) and *EXP* are upregulated during *Arabidopsis* seed germination [79, 80]. Our observations confirmed that a series of cell wall-degrading genes is up-regulated significantly in *P. notoginseng* seeds treated with exogenous GA_3_ (Fig. 8G), and it suggests that exogenous GA_3_ might promote cell wall metabolism and endosperm degradation. Compared with CK, the expression of *EXP* and *PME* were upregulated in *P. notoginseng* seeds treated with LG and HG. Differently, the up-regulated expression level in the seeds treated with HG was significantly higher than those treated with LG (Fig. 7B). Thus, although LG, MG and HG treatments all promote seed germination, to a higher degree HG accelerates the degradation of the cell wall to create more spaces for seed germination by up-regulating *EXP* and *PME*. This could be regarded as the reason for the highest germination rate of *P. notoginseng* seeds treated with HG. In general, we believe that the genes (*LEA*, *LEC1*, *EXP*, *PME* and *XEH*) involved in embryo development and the cell wall degradation might create more spaces for radicle elongation to accelerate the germination in postharvest *P. notoginseng* seeds treated with GA_3_.

## Conclusion

Exogenous GA_3_ increases the content of endogenous hormones GA_3_ through permeation and alters the ratio of GA and ABA to promote seed germination of *P. notogensing*. GA-treated *P. notogensing* seeds maintain higher development and germination than CK treatment. We also find that GA_3_ upregulates DEGs involved in GA biosynthesis and catabolism, embryo development and cell wall loosening, while downregulates ABA biosynthesis and catabolism. Based on the findings of the present work, a model is proposed to explain the dormancy mechanism in recalcitrant *P. notogensing* seeds regulated by GA_3_ (Fig. S8). Exogenous GA_3_ application increases the content of endogenous hormones GA_3_ through permeation to alter the ratio of GA/ABA, and this would contribute to the development of the embryo, break the mechanical constraints of the seed coat and promote the protrusion of the radicle in postharvest recalcitrant seeds. These findings would comprehensively improve our understanding of the potential roles of GA in regulating the dormancy of recalcitrant seeds during the after-ripening process.

## Materials and methods

### Materials and treatments

Seeds were routinely harvested from the plants of 3-year-old *P. notoginseng* (Fig. S9A) that were cultivated at the experimental farm of Wenshan Miao Xiang *P. notoginseng* Industrial Co., Ltd., China (Longitude 104°32′, latitude 23°53′). In November, 3-year-old mature and plump seeds of *P. notoginseng* were selected (Fig. S9B). After artificial peeling (Fig. S9C), the seeds were washed in ddH_2_O, soaked and disinfected with 5% CuSO_4_ bactericidal solution and washed twice, then the seeds were obtained by indoor shade drying out surface moisture. The seeds were submitted to one of three different treatments by soaking for 24 h, the concentrations 50 mg L^−1^ (LG), 250 mg L^−1^ (MG) and 500 mg L^−1^ (HG) of exogenous hormones GA_3_ were selected for soaking treatment, and ddH_2_O-soaked treatment was defined as the control (CK). The soaked seeds with three replicates were placed in a ventilated net basket for 50 days in a sandy stratification chamber at 15 ± 5℃, and sandy humidity at 25% to accomplish after-ripening process. Dark conditions are maintained during after-ripening process with an ambient humidity at 70%. The start point before the seeds were stored in sandy stratification is defined as the time point of 0 days after-ripening (DAR). At each sampling point (0, 30, 50 DAR), the samples from the concentrations of 50 mg L^−1^ (LG) and 500 mg L^−1^ (HG) of GA_3_ were selected based on the results from experiments (Fig. 1) for further assays.

### Microscopic inspection and morphological measurements

A number of seeds were fixed using FAA (70% alcohol: acetic acid: formalin = 18:1:1) for microscopic inspection. The determination of embryo (Em), endosperm (En) and embryo rate (Em/En) was carried out at four time points of 0, 15, 30 and 45 DAR. Seeds were divided in half lengthwise by using a razor blade. Seed sections were examined using a stereoscopic microscope (ZEISS, SteREO Discovery.V20, Germany) equipped with a digital camera. The pictures were processed (brightness and contrast adjusted) and combined using Photoshop CS6 (Adobe, USA).

### HPLC–MS analysis of endogenous hormone GA_3_, ABA and IAA content

Samples used for the determination of endogenous hormone content were frozen in liquid nitrogen at three time points of 0, 30 and 50 DAR. Endogenous hormone ABA, GA_3_ and IAA content in seed of *P. notoginseng* was examined as reported by Pan et al. [81] with some modifications.

### Total RNA extraction and transcriptome analysis

For LG, HG and CK treatment, the sample at 0, 30 and 50 DAR were separated and applied for RNA extraction. Using the Plant Plus Kit (Tiangen, Beijing, China) to extract the total RNA with three replications, and RNA quality was monitored on 1% agarose electrophoresis. RNA purity was checked using the NanoPhotometer® spectrophotometer (IMPLEN, CA, USA), and RNA integrity was assessed using the RNA Nano 6000 Assay Kit of the Bioanalyzer 2100 system (Agilent Technologies, CA, USA). Using NEBNext® UltraTM RNA Library Prep Kit, sequencing libraries were generated for Illumina® (NEB, USA) following the manufacturer’s recommendations and index codes were added to attribute sequences. The libraries were sequenced on an Illumina platform by the Novegene Technology Company (Beijing, China). Raw reads were analyzed and low quality reads and reads containing adapters were removed, resulting in clean reads. The clean reads were mapped to the *P. notogensing* reference genome (Fan et al., 2020) using HISAT2 v2.0.5, and novel genes prediction were made with String Tie (1.3.3b) [82].

Differential expression analysis of paired conditions with three biological replicates per condition was performed using the DESeq2 R package (1.16.1). According to the method of Benjamini and Hochberg [83], the *P*-values were adapted to control the false discovery rate. Gene Ontology (GO) analysis and KEGG analysis [84] of differentially expressed genes (DEGs) were performed by the clusterProfiler R package.

### Gene expression assessment

Gene expression was assayed by Quantitative Real-time PCR (qRT-PCR). The total RNA was isolated from seeds of *P. notogensing* samples using the same scheme described for RNA-Seq. The cDNA was synthesized by using Prime Script RT reagent kit (Takara Bio, Kyoto, Japan). As shown in Table S2, primers were accessed using Premier 3.0 [85] for quantitative PCR (qRT-PCR) and synthesized by Tsingke Biotech Co., Ltd. (Kunming, China). The qRT-PCR reaction was performed with three technical replicates using the Quant studio12 K Flex System (Thermo Fisher Scientific). The *GLYCERALDEHYDE-3-PHOSPHATE DEHYDROGENASE(GAPDH)* was chosen as the internal reference for *P. notoginseng* seeds. Candidate genes were analyzed for relative expression levels using the 2^−ΔΔCt^ algorithm [86] by standardizing their transcript levels of related genes in control. Each sample was analyzed in three replicates.

### Statistical analysis

The experiment was performed in three biological replicates. Statistical analyses were carried out using the SPSS software package (Chicago, IL, USA) and SigmaPlot 10.0, where the variables were present as the mean ± SD (*n* = 3). The least significant difference (LSD) was calculated and *P* < 0.05 was deemed statistically significant. PCA was performed using the prcomp function in R language. Genes with an adjusted *P*-value < 0.05 found by DESeq2 were designated as differential expressions. The terms in GO and KEGG analysis with corrected *P*-value < 0.05 were identified as significant enrichment of differently expressed genes. The gene co-expression network was constructed using the weighted gene co-expression network analysis (WGCNA) package in R language [87].

## Supplementary Information

**Additional file 1:** **Figure S1. **Appearance and morphology of GA_3_-treated *P. notoginseng* seeds after germination. (A) t=30d. (B) t=45d. **Figure S2. **Gene expression distribution. The distribution of gene expression levels for different samples is illustrated by box plots. The X-axis represents sample names, the Y-axis on the left indicates the log_2_ (FPKM + 1). The Box plots for each region are plotted against five statistics (maximum, upper quartile, median, lower quartile and minimum). **Figure S3.** Pearson correlation analysis of gene expression levels between samples. The X-axis and Y-axis in the graph are the squares of the correlation coefficients for each sample. **Figure S4. **Venn diagrams of DEGs. (A) DEGs between the control and Low concentration GA_3_-treated (LG) *P. notoginseng* seeds during the after-ripening process. (B) DEGs between the control and the High concentration GA_3_-treated (HG) *P. notoginseng* seeds during after-ripening process. **Figure S5. **GO analysis of differentially expressed genes (DEGs) in control and GA_3_-treated *P. notoginseng* seeds during after-ripening process. (A) Top 30 most enriched GO terms of DEGs between CK_30 VS LG_30. (B) Top 30 most enriched GO terms of DEGs between CK_30 VS HG_30. The Y-axis on the left represents GO terms, including biological process, cellular component, and molecular function, the X-axis indicates genes number of each term. Up-regulated genes are shown in red bar, and down-regulated genes are shown in blue bar. **Figure S6. **KEGG analysis of differentially expressed genes (DEGs) in control and GA_3_-treated *P. notoginseng* seeds during after-ripening process. (A) Top 20 most enriched KEGG pathways of DEGs between CK_0 vs LG_0. (B) Top 20 most enriched KEGG pathways of DEGs between CK_0 vs HG_0. The Y-axis on the left represents GO KEGG pathways, the X-axis indicates the “Gene Ratio” represented by the ratio of DEGs numbers to total annotated gene numbers of each pathway. Low *P* values are shown in the red circle, and high P values are shown in the purple circle. The area of a circle represents DEGs number. **Figure S7. **WGCNA network module mining. (A) Clustering dendrogram of genes. In the dendrogram, each leaf corresponds to a gene. A total of 21,988 genes resulted in 22 co-expression modules labelled by different merged colors. (B) Scatterplots of Gene Significance for Em/En vs Module Membership in the coral2 and black (D) modules. (C) Scatterplots of Gene Significance for germination vs Module Membership in the coral2 and black (E) modules. **Figure S8.** A model for the possible mechanism of exogenous GA_3_ regulation of germination in *P. notoginseng* seeds during the after-ripening process at the transcriptional levels. Exogenous GA_3_ application increases the content of endogenous hormones GA_3_ through permeation, and this alter would contribute to the expression of genes in embryo development, cell wall relaxation and ABA signal transduction, consequently shortening after-ripening process and promoting recalcitrant seed germination. Genes marked in red indicate that these genes were GA-induced, and likewise, black ones suggest that the genes were GA-repressed. Arrows and blunted lines designate positive and inhibitory interactions, respectively. **Figure S9.** Appearance and morphology of 3-year-old *P. notoginseng* seeds. (A) Seeds are routinely harvested from the 3-year-old *P. notoginseng.* (B) Mature and plump seeds of *P. notoginseng* before artificial peeling. (C) The morphology of *P. notoginseng* seeds after artificial peeling.

**Additional file 2: Table S1. **Evaluation of sample sequencing data. **Table S2. **List of primers used in qRT-PCR analysis.

## Data Availability

The raw sequencing data from this study have been deposited in the Genome Sequence Archive in BIG Data Center (https://bigd.big.ac.cn/), Beijing Institute of Genomics (BIG), Chinese Academy of Sciences, under the accession number: CRA008378. Other data generated or analyzed during this study are included in this published article and its supplementary information files. Hoo & Tseng first undertook the formal identification of the plant material *Panax notoginseng* (Burkill) (Journal of Systematics and Evolution 11: 435, 1973) in Flora of China.

## Abbreviations

*ABI5*: ABA-INSENSITIVE5
*CPS*: Ent-copalyl diphosphate synthase
DAR: Days after-ripening
*EXP*: Expansin
Em/En: Embryo rate
*GA20ox*: GA 20-oxidase
*LEA*: Late Embyogenesis Abundant
*LEC1*: Leafy Cntyledon1
*PYL*: Pyrabactin resistance 1-like
*PME*: Pectinesterase
*PP2C*: Protein Phosphatase 2C
