# Supplementary material for: Exogenous gibberellic acid shortening after-ripening process and promoting seed germination in a medicinal plant Panax notoginseng
Source: BMC Plant Biol. 2023 Feb 1;23:67. doi: 10.1186/s12870-023-04084-3 (PMC9890714; doi:10.1186/s12870-023-04084-3)
Supplement: Supplementary file 1 — Additional file 1: Figure S1. Appearance and morphology of GA3-treated P. notoginseng seeds after germination. (A) t=30d. (B) t=45d. Figure S2. Gene expression distribution. The distribution of gene expression levels for different samples is illustrated by box plots. The X-axis represents sample names, the Y-axis on the left indicates the log2 (FPKM + 1). The Box plots for each region are plotted against five statistics (maximum, upper quartile, median, lower quartile and minimum). Figure S3. Pearson correlation analysis of gene expression levels between samples. The X-axis and Y-axis in the graph are the squares of the correlation coefficients for each sample. Figure S4. Venn diagrams of DEGs. (A) DEGs between the control and Low concentration GA3-treated (LG) P. notoginseng seeds during the after-ripening process. (B) DEGs between the control and the High concentration GA3-treated (HG) P. notoginseng seeds during after-ripening process. Figure S5. GO analysis of differentially expressed genes (DEGs) in control and GA3-treated P. notoginseng seeds during after-ripening process. (A) Top 30 most enriched GO terms of DEGs between CK_30 VS LG_30. (B) Top 30 most enriched GO terms of DEGs between CK_30 VS HG_30. The Y-axis on the left represents GO terms, including biological process, cellular component, and molecular function, the X-axis indicates genes number of each term. Up-regulated genes are shown in red bar, and down-regulated genes are shown in blue bar. Figure S6. KEGG analysis of differentially expressed genes (DEGs) in control and GA3-treated P. notoginseng seeds during after-ripening process. (A) Top 20 most enriched KEGG pathways of DEGs between CK_0 vs LG_0. (B) Top 20 most enriched KEGG pathways of DEGs between CK_0 vs HG_0. The Y-axis on the left represents GO KEGG pathways, the X-axis indicates the “Gene Ratio” represented by the ratio of DEGs numbers to total annotated gene numbers of each pathway. Low P values are shown in the red circle, and high P v [file 12870_2023_4084_MOESM1_ESM.docx]

**Figure S1** Appearance and morphology of GA_3_-treated *P. notoginseng* seeds after germination. (A)t=30d. (B) t=45d.


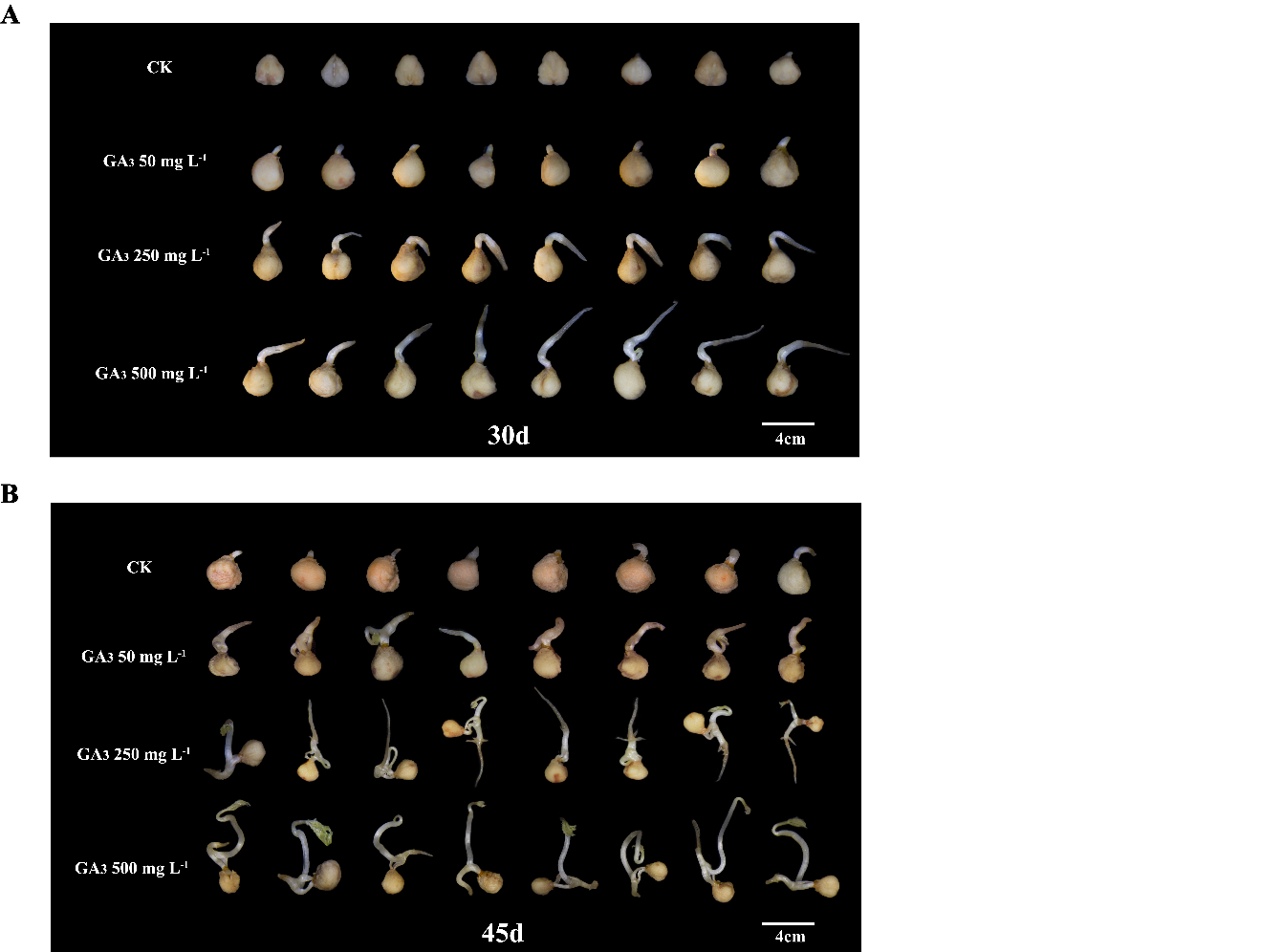


**Figure S2** Gene expression distribution. The distribution of gene expression levels for different samples is illustrated by box plots. The X-axis represents sample names, the Y-axis on the left indicates the log_2_ (FPKM + 1). The Box plots for each region are plotted against five statistics (maximum, upper quartile, median, lower quartile and minimum).

**
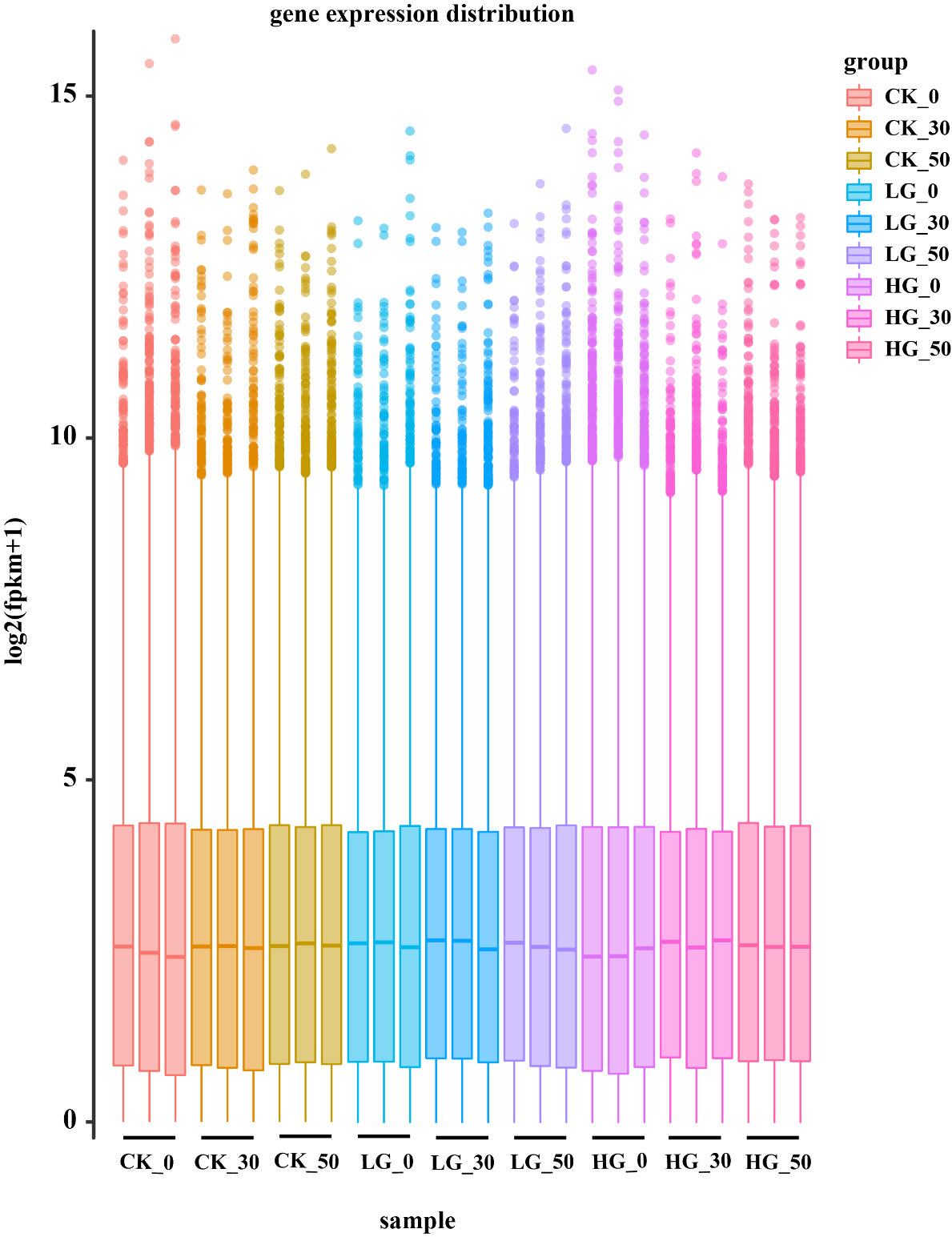
**

**Figure S3** Pearson correlation analysis of gene expression levels between samples. The X-axis and Y-axis in the graph are the squares of the correlation coefficients for each sample.

**
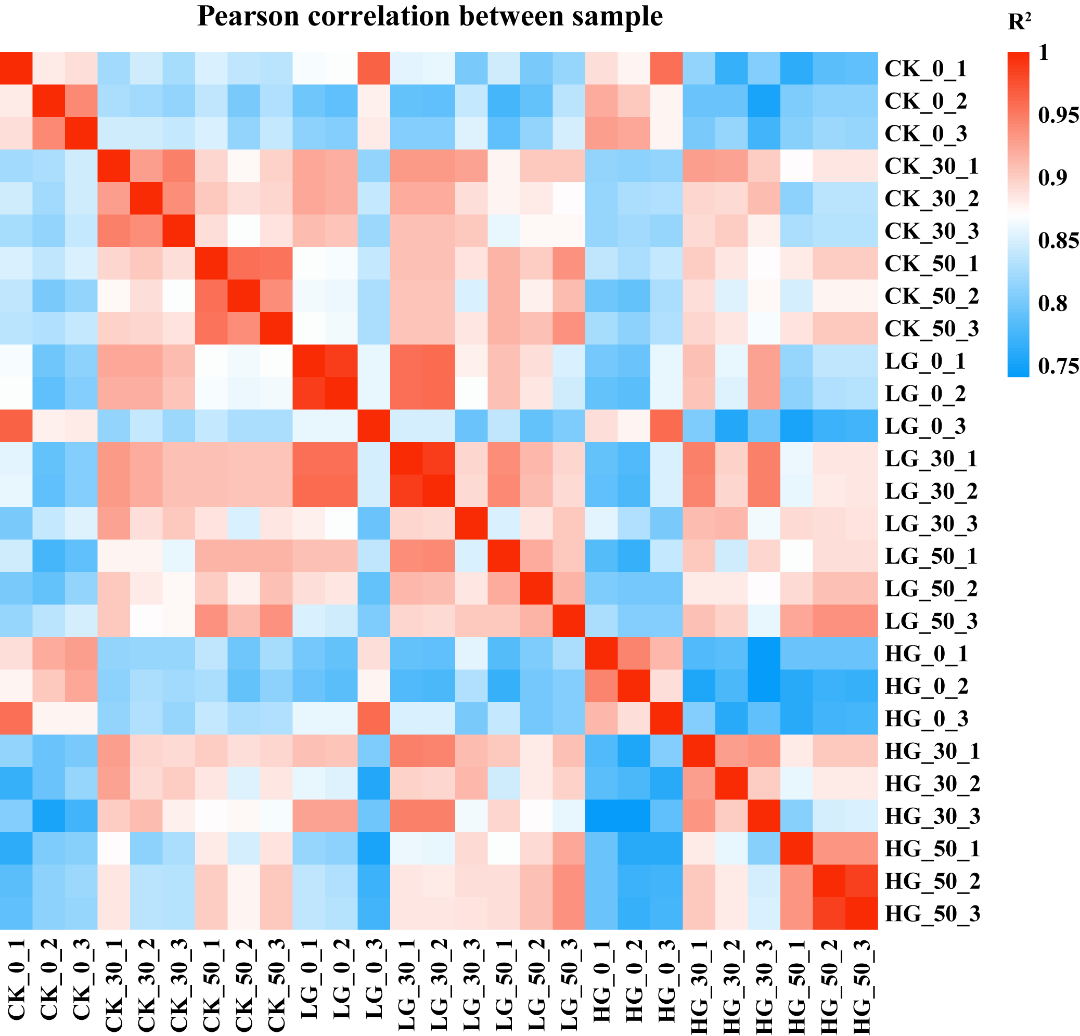
**

**Figure S4** Venn diagrams of DEGs. (A) DEGs between the control and Low concentration GA_3_-treated (LG) *P. notoginseng* seeds during the after-ripening process. (B) DEGs between the control and the High concentration GA_3_-treated (HG) *P. notoginseng* seeds during after-ripening process.

**
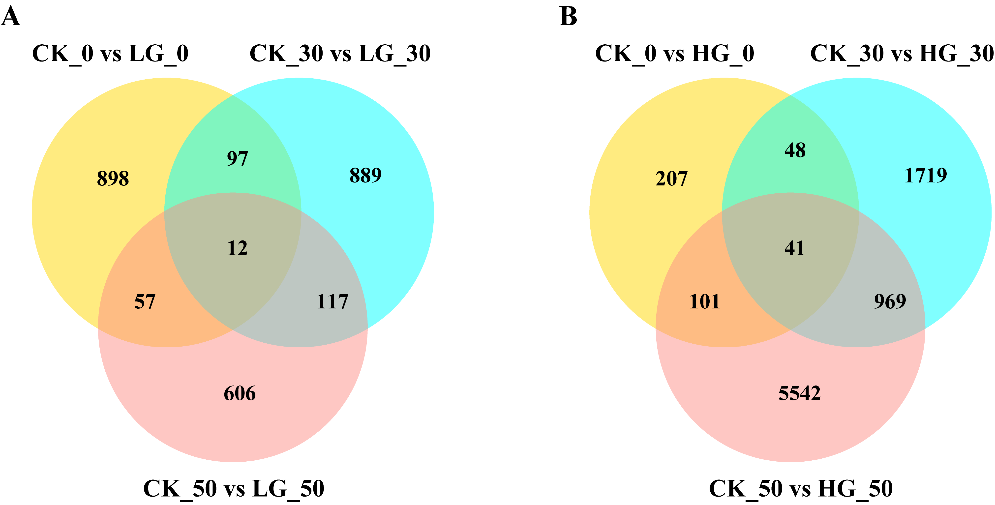
**

**Figure S5** GO analysis of differentially expressed genes (DEGs) in control and GA_3_-treated *P. notoginseng* seeds during after-ripening process. (A) Top 30 most enriched GO terms of DEGs between CK_30 VS LG_30. (B) Top 30 most enriched GO terms of DEGs between CK_30 VS HG_30. The Y-axis on the left represents GO terms, including biological process, cellular component, and molecular function, the X-axis indicates genes number of each term. Up-regulated genes are shown in red bar, and down-regulated genes are shown in blue bar.

**
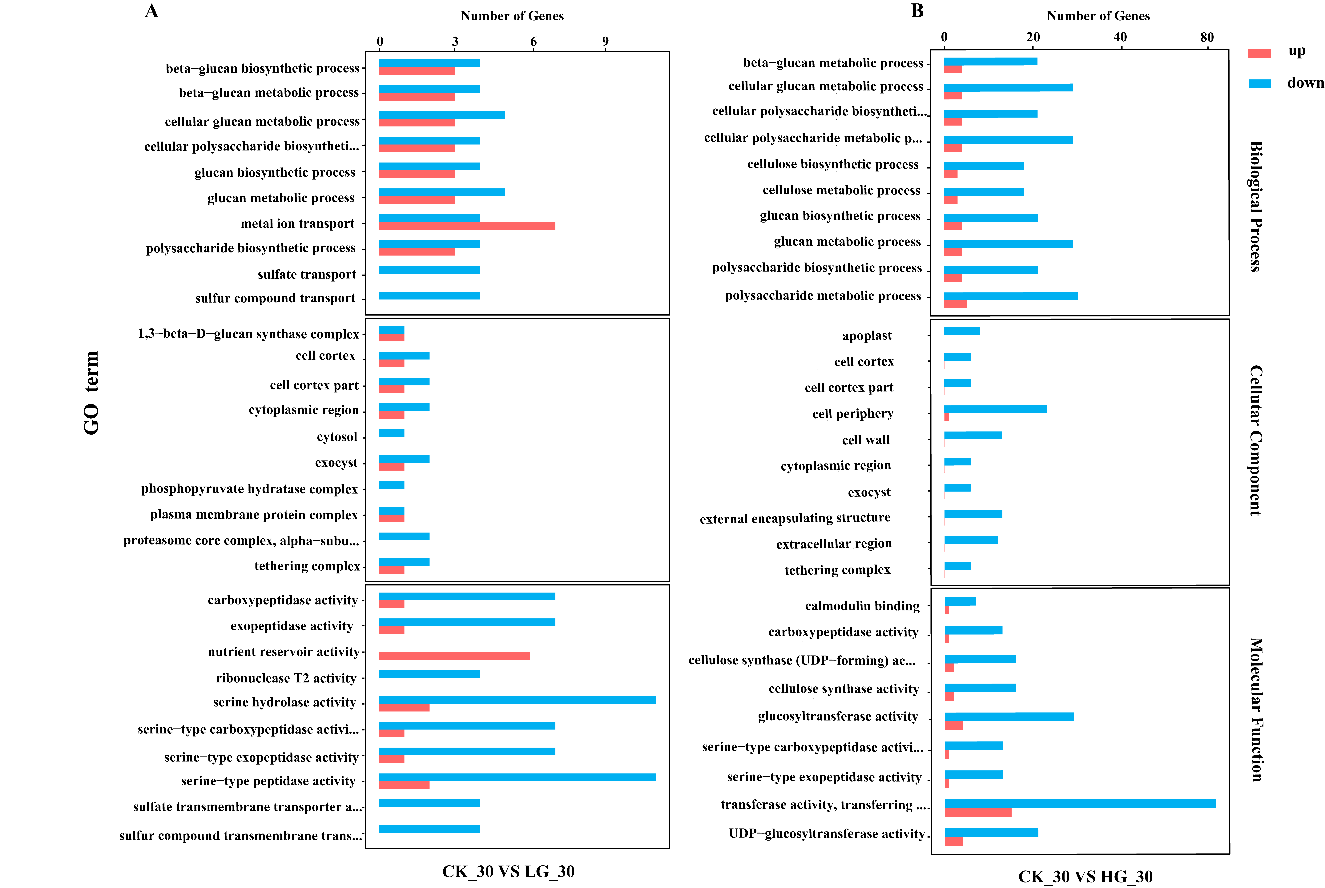
**

**Figure S6** KEGG analysis of differentially expressed genes (DEGs) in control and GA_3_-treated *P. notoginseng* seeds during after-ripening process. (A) Top 20 most enriched KEGG pathways of DEGs between CK_0 vs LG_0. (B) Top 20 most enriched KEGG pathways of DEGs between CK_0 vs HG_0. The Y-axis on the left represents GO KEGG pathways, the X-axis indicates the “Gene Ratio” represented by the ratio of DEGs numbers to total annotated gene numbers of each pathway. Low P values are shown in the red circle, and high P values are shown in the purple circle. The area of a circle represents DEGs number.

**
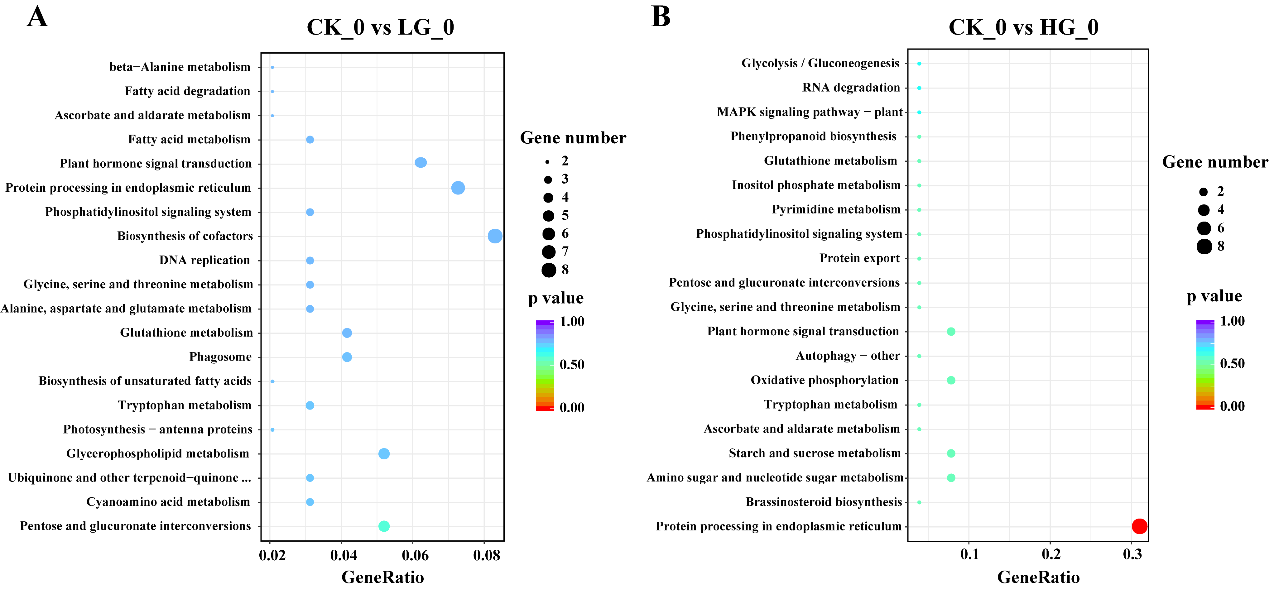
**

**Figure S7** WGCNA network module mining. (A) Clustering dendrogram of genes. In the dendrogram, each leaf corresponds to a gene. A total of 21,988 genes resulted in 22 co-expression modules labelled by different merged colors. (B) Scatterplots of Gene Significance for Em/En vs Module Membership in the coral2 and black (D) modules. (C) Scatterplots of Gene Significance for germination vs Module Membership in the coral2 and black (E) modules.

**
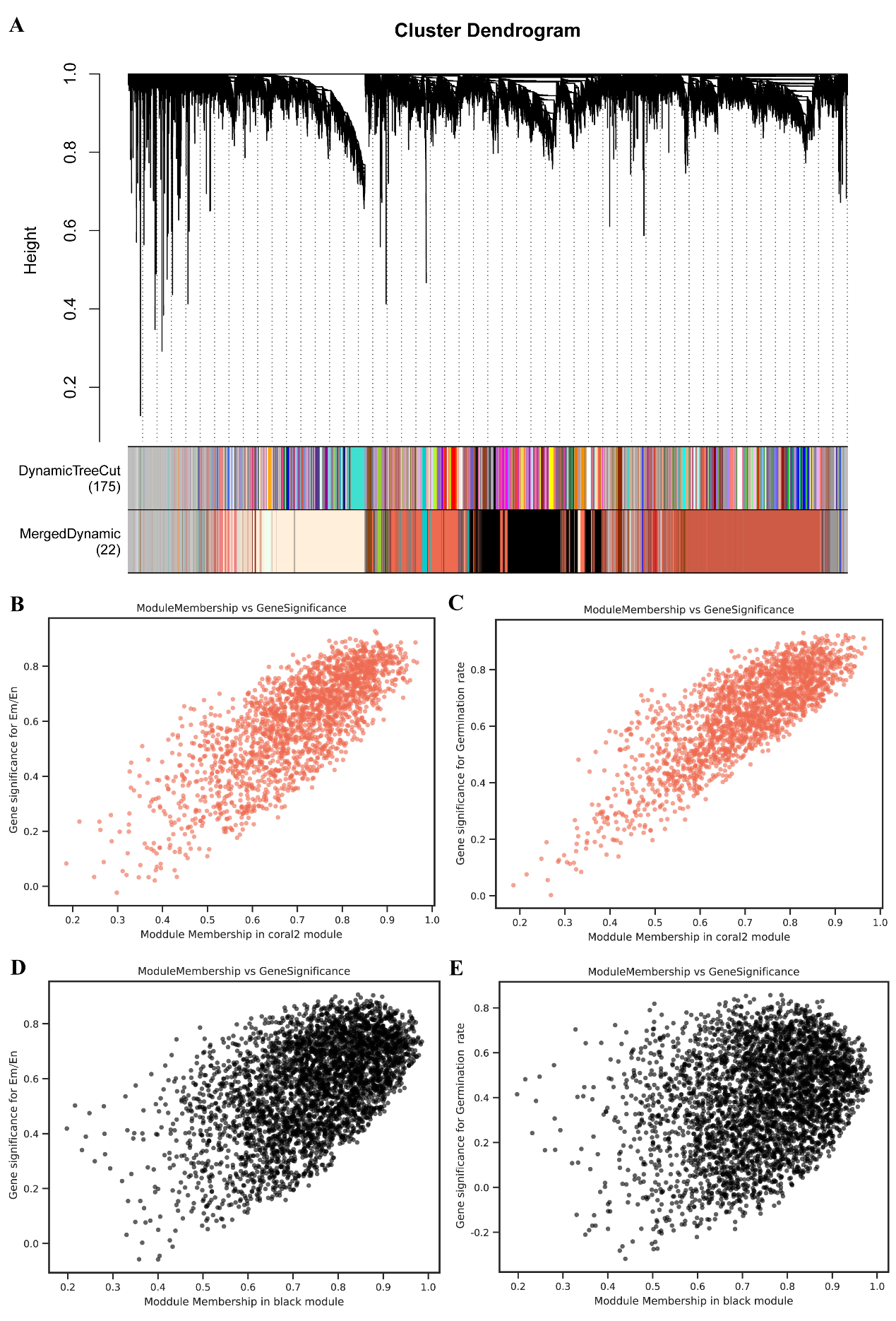
**

**Figure S8** A model for the possible mechanism of exogenous GA_3_ regulation of germination in *P. notoginseng* seeds during the after-ripening process at the transcriptional levels. Exogenous GA_3_ application increases the content of endogenous hormones GA_3_ through permeation, and this alter would contribute to the expression of genes in embryo development, cell wall relaxation and ABA signal transduction, consequently shortening after-ripening process and promoting recalcitrant seed germination. Genes marked in red indicate that these genes were GA-induced, and likewise, black ones suggest that the genes were GA-repressed. Arrows and blunted lines designate positive and inhibitory interactions, respectively.


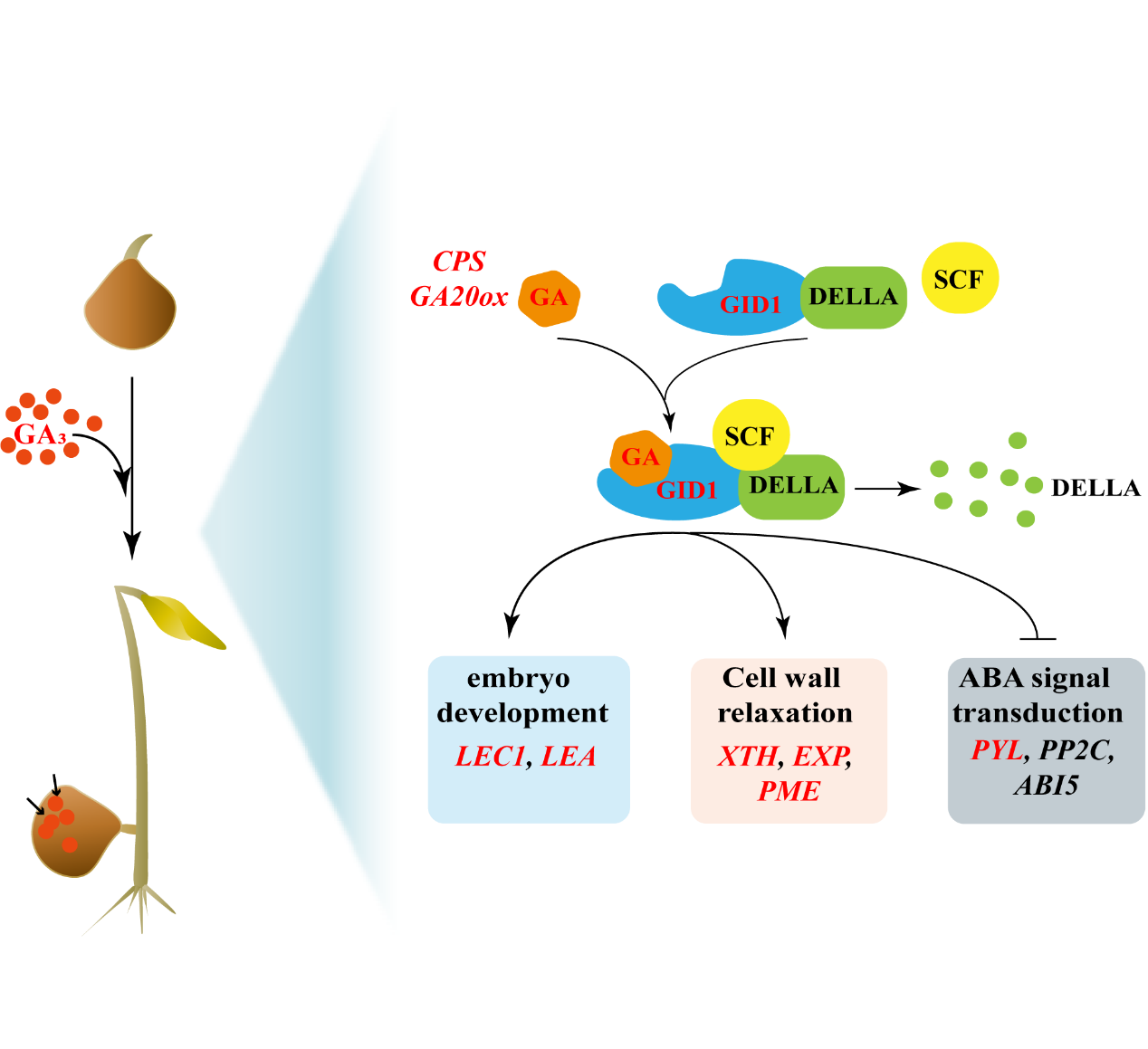


**Figure S9** Appearance and morphology of 3-year-old *P. notoginseng* seeds. (A) Seeds are routinely harvested from the 3-year-old *P. notoginseng.* (B) Mature and plump seeds of *P. notoginseng* before artificial peeling. (C) The morphology of *P. notoginseng* seeds after artificial peeling.

**
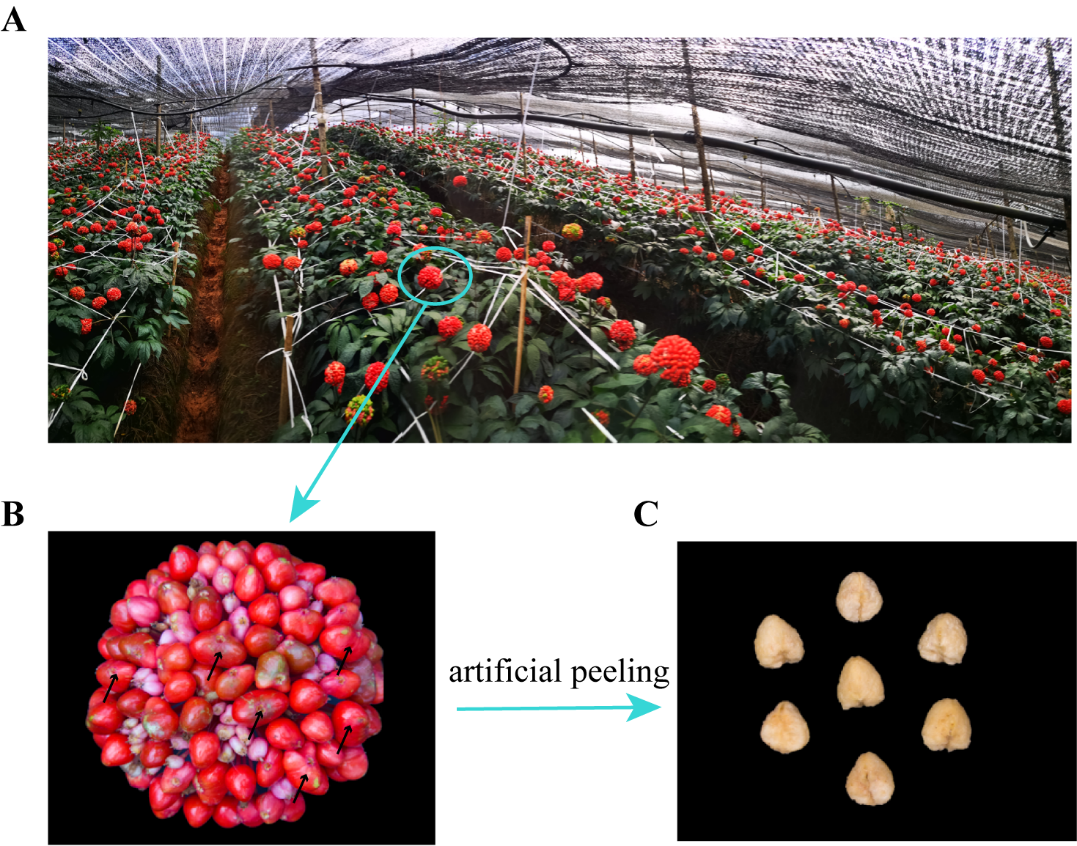
**
