# Supplementary material for: Exogenous gibberellic acid shortening after-ripening process and promoting seed germination in a medicinal plant Panax notoginseng
Source: BMC Plant Biol. 2023 Feb 1;23:67. doi: 10.1186/s12870-023-04084-3 (PMC9890714; doi:10.1186/s12870-023-04084-3)
Supplement: Supplementary file 2 — Additional file 2: Table S1. Evaluation of sample sequencing data. Table S2. List of primers used in qRT-PCR analysis. [file 12870_2023_4084_MOESM2_ESM.docx]

**Table S1 Evaluation of sample sequencing data.**

| sample | raw reads number | clean reads number | clean bases | error rate  (%) | clean Q20 bases rate  (%) | clean Q30 bases rate  (%) |
| --- | --- | --- | --- | --- | --- | --- |
| CK_0_1 | 47554084 | 46815702 | 7.02G | 0.02 | 98.02 | 94.08 |
| CK_0_2 | 47956860 | 46919124 | 7.04G | 0.02 | 97.60 | 93.17 |
| CK_0_3 | 47069976 | 45916162 | 6.89G | 0.02 | 97.65 | 93.29 |
| LG_0_1 | 47452772 | 46769434 | 7.02G | 0.02 | 97.82 | 93.58 |
| LG_0_2 | 45339512 | 44727404 | 6.71G | 0.02 | 98.01 | 94.08 |
| LG_0_3 | 43929674 | 43111468 | 6.47G | 0.02 | 97.87 | 93.72 |
| HG_0_1 | 47538852 | 46684610 | 7.00G | 0.02 | 98.04 | 94.14 |
| HG_0_2 | 46480934 | 45715822 | 6.86G | 0.02 | 97.57 | 93.11 |
| HG_0_3 | 52689372 | 51812850 | 7.77G | 0.02 | 97.97 | 93.97 |
| CK_30_1 | 47988046 | 46770276 | 7.02G | 0.02 | 97.73 | 93.39 |
| CK_30_2 | 47121260 | 46087118 | 6.91G | 0.02 | 97.95 | 93.92 |
| CK_30_3 | 52246764 | 50775950 | 7.62G | 0.02 | 97.47 | 92.88 |
| LG_30_1 | 45265548 | 44439024 | 6.67G | 0.02 | 97.94 | 93.85 |
| LG_30_2 | 47477116 | 46805200 | 7.02G | 0.02 | 97.95 | 93.84 |
| LG_30_3 | 45327656 | 44743018 | 6.71G | 0.02 | 97.40 | 92.73 |
| HG_30_1 | 48849000 | 48011460 | 7.20G | 0.02 | 97.63 | 93.15 |
| HG_30_2 | 48014394 | 47257916 | 7.09G | 0.02 | 98.08 | 94.28 |
| HG_30_3 | 47056682 | 46156508 | 6.92G | 0.02 | 97.84 | 93.66 |
| CK_50_1 | 47184604 | 46450880 | 6.97G | 0.02 | 97.87 | 93.80 |
| CK_50_2 | 45327334 | 44037090 | 6.61G | 0.02 | 97.70 | 93.36 |
| CK_50_3 | 50521810 | 49358510 | 7.40G | 0.02 | 97.85 | 93.65 |
| LG_50_1 | 46724874 | 45946054 | 6.89G | 0.02 | 97.84 | 93.70 |
| LG_50_2 | 45916154 | 45088374 | 6.76G | 0.02 | 98.08 | 94.25 |
| LG_50_3 | 45967536 | 45204254 | 6.78G | 0.02 | 97.94 | 93.90 |
| HG_50_1 | 45519978 | 44787148 | 6.72G | 0.02 | 97.95 | 93.95 |
| HG_50_2 | 49238306 | 48294500 | 7.24G | 0.02 | 97.82 | 93.64 |
| HG_50_3 | 43582536 | 42958006 | 6.44G | 0.02 | 97.56 | 93.08 |

**Table S2** List of primers used in qRT-PCR analysis.

| Gene ID | Gene name | Primer sequence 5'→3' | | Size/bp |
| --- | --- | --- | --- | --- |
|  |  | Forward | Reverse |  |
| PN000879 | *CPS* | CCGCATTACAACGCACGAAT | AAGTGCATGAACGACTTGCG | 20 |
| PN021274 | *GA20ox* | AAAGGTCCTGCTGTTGGGAC | GGCATCTCCAGCAGGATTGT | 20 |
| PN037246 | *DELLA* | AGACACCAGAGTTGACGAGC | TGACCCATCTCAAGTCGTCG | 20 |
| PN005781 | *DELLA* | TAACTGGTATTGGACCGCCG | ACGGAAGCCATCACCTTCTC | 20 |
| PN019190 | *PYL* | TCGAAGGTACCACAAGCACG | ACCACCTACAATCCTGACGC | 20 |
| PN037624 | *ABI5* | AGTGCATCGGAAGTGACTGAG | GTCCTCCGCATTGTTTTAGGC | 21 |
| PN010244 | *PME* | TAACTGCTCAAGGCCGAACC | CGGTGAATTTAGCCGCATCC | 20 |
| PN015405 | *LEA* | ACGACAAGATCGAAGCTCGG | CAACGCCTCCAATCCCAATC | 20 |
| PN009353 | *GAPDH* | TGGAATGGCCTTCCGAGTTC | CGTACCACGCGACAAGTTTC | 20 |

*GADPH* was used as the reference one. C*PS*: ent-copalyl diphosphate synthase; *GA20ox*: GA20-oxidase; *PYL*: Pyrabactin resistance 1-like; *ABI5*: ABA-INSENSITIVE5; *PME*: Pectinesterase; *LEA*: Late Embryogenesis Abundant; *GAPDH*: GLYCERALDEHYDE-3-PHOSPHATE DEHYDROGENASE.
